# Tropical Logistic Regression Model on Space of Phylogenetic Trees

**DOI:** 10.1007/s11538-024-01327-8

**Published:** 2024-07-02

**Authors:** Georgios Aliatimis, Ruriko Yoshida, Burak Boyacı, James A. Grant

**Affiliations:** 1 STOR-i Centre for Doctoral Training, Lancaster University, Lancaster, LA1 4YW UK; 2 Department of Operations Research, Naval Postgraduate School, 1411 Cunningham Road, Monterey, CA 93943 USA; 3 Management School, Lancaster University, Lancaster, LA1 4YX UK; 4 Department of Mathematics and Statistics, Lancaster University, Lancaster, LA1 4YX UK

**Keywords:** Coalescent model, Classifications, Gene trees and species trees, Tropical geometry, Ultrametrics

## Abstract

Classification of gene trees is an important task both in the analysis of multi-locus phylogenetic data, and assessment of the convergence of Markov Chain Monte Carlo (MCMC) analyses used in Bayesian phylogenetic tree reconstruction. The logistic regression model is one of the most popular classification models in statistical learning, thanks to its computational speed and interpretability. However, it is not appropriate to directly apply the standard logistic regression model to a set of phylogenetic trees, as the space of phylogenetic trees is non-Euclidean and thus contradicts the standard assumptions on covariates. It is well-known in tropical geometry and phylogenetics that the space of phylogenetic trees is a tropical linear space in terms of the max-plus algebra. Therefore, in this paper, we propose an analogue approach of the logistic regression model in the setting of tropical geometry. Our proposed method outperforms classical logistic regression in terms of Area under the ROC Curve in numerical examples, including with data generated by the multi-species coalescent model. Theoretical properties such as statistical consistency have been proved and generalization error rates have been derived. Finally, our classification algorithm is proposed as an MCMC convergence criterion for Mr Bayes. Unlike the convergence metric used by Mr Bayes which is only dependent on tree topologies, our method is sensitive to branch lengths and therefore provides a more robust metric for convergence. In a test case, it is illustrated that the tropical logistic regression can differentiate between two independently run MCMC chains, even when the standard metric cannot.

## Introduction

Phylogenomics is a new field that applies tools from phylogenetics to genome datasets. The multi-species coalescent model is often used to model the distribution of gene trees under a given species tree (Maddison 2008). The first step in statistical analysis of phylogenomic data is to analyze sequence alignments to determine whether their evolutionary histories are congruent with each other. In this step, evolutionary biologists aim to identify genes with unusual evolutionary events, such as duplication, horizontal gene transfer, or hybridization (Ané et al. 2007). To accomplish this, they compare multiple sets of *gene trees*, that is, phylogenetic trees reconstructed from alignments of genes, with each gene tree characterised by the aforementioned evolutionary events. The classification of gene trees into different categories is therefore important for analyzing multi-locus phylogenetic data.

Tree classification can also help in assessing the convergence of Markov Chain Monte Carlo (MCMC) analyses for Bayesian inference on phylogenetic tree reconstruction. Often, we apply MCMC samplers to estimate the posterior distribution of a phylogenetic tree given an observed alignment. These samplers typically run multiple independent Markov chains on the same observed dataset. The goal is to check whether these chains converge to the same distribution. This process is often done by comparing summary statistics computed from sampled trees. These statistics often only depend on the tree topologies, and so they naturally lose information about the branch lengths of the sampled trees. Alternatively, we propose the use of a classification model that classifies trees from different chains and uses statistical measures such as the Area under the ROC Curve (AUC) to indicate how distinguishable the two chains are. Consequently, high values of AUCs indicate that the chains have not converged to the equilibrium distribution. Currently, there is no classification model over the space of phylogenetic trees, the set of all possible phylogenetic trees with a fixed number of leaves. In this paper, we propose a classifier that is appropriate for the tree space and is sensitive to branch lengths, unlike the summary statistics of most MCMC convergence diagnostic tools.

In Euclidean geometry, the logistic regression model is the simplest generalized linear model for classification. It is a supervised learning method that classifies data points by modeling the log-odds of having a response variable in a particular class as a linear combination of predictors. This model is very popular in statistical learning due to its simplicity, computational speed and interpretability. However, directly applying such classical supervised models to a set of sampled trees may be misleading, since the space of phylogenetic trees does not conform to Euclidean geometry.

The space of phylogenetic trees with labeled leaves [*m*] is a union of lower dimensional polyhedral cones with dimension $m - 1$ over $\mathbb {R}^e$ where $$e = \left( {\begin{array}{c}m\\ 2\end{array}}\right) $$ (Speyer and Sturmfels 2009; Lin et al. 2017). This space is not Euclidean and even lacks convexity (Lin et al. 2017). In fact, Speyer and Sturmfels (2009) showed that the space of phylogenetic trees is a *tropicalization* of linear subspaces defined by a system of tropical linear equations (Page et al. 2020) and is therefore a tropical linear space.

Consequently, many researchers have applied tools from tropical geometry to statistical learning methods in phylogenomics, such as principal component analysis over the space of phylogenetic trees with a given set of leaves [*m*] (Page et al. 2020; Yoshida et al. 2019), kernel density estimation (Yoshida et al. 2022a), MCMC sampling (Yoshida et al. 2022b), and support vector machines (Yoshida et al. 2021). Recently, Akian et al. (2021) proposed a tropical linear regression over the tropical projective space as the best-fit tropical hyperplane. However, our logistic regression model is built from first principles and is not a trivial extension of the aforementioned tropical regression model.

In this paper, an analog of the logistic regression is developed over the tropical projective space, which is the quotient space $\mathbb {R}^e/\mathbb {R}{} \textbf{1}$ where $\textbf{1}:= (1, 1, \ldots , 1)$. Given a sample of observations within this space, the proposed model finds the “best-fit” tree representative $\omega _Y \in \mathbb {R}^e/\mathbb {R}{} \textbf{1}$ of each class $Y \in \{0,1\}$ and the “best-fit” deviation of the gene trees. This tree representative is a statistical parameter and can be interpreted as the corresponding species tree of the gene trees. The deviation parameter is defined in terms of the variability of branch lengths of gene trees. It is established that the median tree, specifically the Fermat–Weber point, can asymptotically approximate the inferred tree representative of each class. The response variable $Y \in \{0,1\}$ has conditional distribution $Y|X \sim \textrm{Bernoulli}( S(h(X)))$, where *h*(*x*) is small when *x* is close to $\omega _0$ and far away from $\omega _{1}$ and vice versa.

In Sect. 1 an overview of tropical geometry and its connections to phylogenetics is presented. The one-species and two-species tropical logistic models are developed in Sect. 2. Theoretical results, including the optimality of the proposed method over tropically distributed predictor trees, the distance distribution of those trees from their representative, the consistency of estimators and the generalization error of each model are stated in Sect. 2 and proved in “Appendix A”. Section 3 explains the benefit and suitability of using the Fermat–Weber point approximation for the inferred trees and a sufficient optimality condition is stated. Computational results are presented in Sect. 4 where a toy example is considered for illustration purposes. Additionally, a comparison study between classical, tropical and BHV logistic regression is conducted on data generated under the coalescent model. In both the toy example and the coalescent gene trees example, our model outperforms the alternative regression models. Finally, our model is proposed as an alternative MCMC convergence criterion in Sect. 4.3. The paper concludes with a discussion in Sect. 5. The code developed and implemented for the proposed model can be found in Aliatimis (2024).

The dataset can be found at DRYAD with DOI: 10.5061/dryad.tht76hf65.

## Tropical Geometry and Phylogenetic Trees

### Tropical Basics

This section covers the basics of tropical geometry and provides the theoretical background for the model developed in later sections. The concept of a tropical metric will be used when defining a suitable distribution for the gene trees. For more details regarding the basic concepts of tropical geometry covered in this section, readers are recommended to consult Maclagan and Sturmfels (2015).

A key tool from tropical geometry is the *tropical metric* also known as the *tropical distance* defined as follows:

#### Definition 1

(*Tropical distance*) The *tropical distance*, more formally known as the Generalized Hilbert projective metric, between two vectors $v, \, w \in ({\mathbb {R}}\cup \{-\infty \})^e$ is defined as

1 $$\begin{aligned} d_\textrm{tr}(v,w) := \Vert v-w \Vert _\textrm{tr} = \max _{i} \bigl \{ v_i - w_i \bigr \} - \min _{i} \bigl \{ v_i - w_i \bigr \}, \end{aligned}$$

where $$v = (v_1, \ldots , v_e)$$ and $$w= (w_1, \ldots , w_e)$$.

#### Remark 1

Consider two vectors $v=(c,\dots ,c) = c \textbf{1} \in {\mathbb {R}}^e$ and $w=\textbf{0} \in {\mathbb {R}} ^ e$. It is easy to verify that $d_\textrm{tr}(v,w) = 0$ and as a result $d_\textrm{tr}$ is not a metric in ${\mathbb {R}} ^ e$. The space in which $d_\textrm{tr}$ is a metric treats all points in $\{ c \textbf{1}: c \in {\mathbb {R}} \} = \mathbb {R} \textbf{1}$ as the same point. The quotient space $( {\mathbb {R}} \cup \{-\infty \} ) ^ e / {\mathbb {R}} \textrm{1}$ achieves just that.

#### Proposition 1

The function $d_\textrm{tr}$ is a well-defined metric on $({\mathbb {R}}\cup \{-\infty \})^e \!/{\mathbb {R}} \textbf{1},$ where $\textbf{1} \in {\mathbb {R}} ^ e$ is the vector of all-ones.

### Equidistant Trees and Ultrametrics

Phylogenetic trees depict the evolutionary relationship between different taxa. For example, they may summarise the evolutionary history of certain species. The leaves of the tree correspond to the species studied, while internal nodes represent (often hypothetical) common ancestors of those species and their ancestors. In this paper, only rooted phylogenetic trees are considered, with the common ancestor of all taxa based on the root of the tree. The branch lengths of these trees are measured in evolutionary units, i.e. the amount of evolutionary change. Under the molecular clock hypothesis, the rate of genetic change between species is constant over time, which implies genetic equidistance and allows us to treat evolutionary units as proportional to time units. Consequently, phylogenetic trees of extant species are *equidistant trees*.

#### Definition 2

(*Equidistant tree*) Let *T* be a rooted phylogenetic tree with leaf label set [*m*], where $m \in \mathbb {N}$ is the number of leaves. If the distance from all leaves $i \in [m]$ to the root is the same, then *T* is an equidistant tree.

It is noted that the molecular clock hypothesis has limitations and the rate of genetic change can in fact vary from one species to another. However, the assumption that gene trees are equidistant is not unusual in phylogenomics; the multispecies coalescent model makes that assumption in order to conduct inference on the species tree from a sample of gene trees (Maddison and Maddison 2009). The proposed classification method is not restricted to equidistant trees, but all coalescent model gene trees produced in Sect. 4.2. are equidistant.

To conduct any mathematical analysis, a vector representation of trees is needed. A common way is to use BHV coordinates (Billera et al. 2001) but in this paper *distance matrices* are used instead, which are then transformed into vectors. The main reason is simplicity and computational efficiency; it is much easier to compute gradients in the tropical projective torus than in the BHV space.

#### Definition 3

(*Distance matrix*) Consider a phylogenetic tree *T* with leaf label set [*m*]. Its distance matrix $D \in \mathbb {R}^{m \times m}$ has components $D_{ij}$ being the pairwise distance between a leaf $i \in [m]$ to a leaf $j \in [m]$. It follows that the matrix is symmetric with zeros on its diagonals. For equidistant trees, $D_{ij}$ is equal to twice the difference between the current time and the latest time that the common ancestor of *i* and *j* was alive.

To form a vector, the distance matrix *D* is mapped onto $\mathbb {R}^e$ by vectorizing the strictly upper triangular part of *D*, i.e.

$$\begin{aligned} D \mapsto (D_{12}, \dots , D_{1m}, D_{23}, \dots , D_{2m}, \dots , D_{(m-1)m}) \in \mathbb {R}^e, \end{aligned}$$

where the dimension of the resulting vector is equal to the number of all possible pairwise combinations of leaves in *T*. Hence the dimension of the phylogenetic tree space is $$e = \left( {\begin{array}{c}m\\ 2\end{array}}\right) $$. In what follows, the connection between the space of phylogenetic trees and tropical linear spaces is established.

#### Definition 4

(*Ultrametric*) Consider the distance matrix $D \in \mathbb {R}^{m \times m}$. Then if

$$\begin{aligned} \max \{D_{ij}, D_{jk}, D_{ik}\} \end{aligned}$$

is attained at least twice for any $i,j,k \in [m]$, *D* is an *ultrametric*. Note that the distance map $d(i,j) = D_{ij}$ forms a metric in [*m*], with the strong triangular inequality satisfied. The space of ultrametrics is denoted as $\mathcal {U}_m$.

#### Theorem 1

(noted in Buneman (1974)) Suppose we have an equidistant tree *T* with a leaf label set [*m*] and *D* as its distance matrix. Then, *D* is an ultrametric if and only if *T* is an equidistant tree.

Using Theorem 1, if we wish to consider all possible equidistant trees, then it is equivalent to consider the space of ultrametrics as the space of phylogenetic trees on [*m*]. Here we define $\mathcal {U}_m$ as the space of ultrametrics with a set of leaf labels [*m*]. Theorem 5 (explained in Ardila and Klivans 2006; Page et al. 2020) in “Appendix B” establishes the connection between phylogenetic trees and tropical geometry by stating that the ultrametric space is a tropical linear space.

## Method

Our logistic regression model is designed to capture the association between a binary response variable $Y\in \{0,1\}$ and an explanatory variable vector $X\in \mathbb {R}^n$, where *n* is the number of covariates in the model. Under the logistic model, $Y \sim \text {Bernoulli}(p(x|\omega ))$ where

2 $$\begin{aligned} p(x|\omega ) = \mathbb {P}(Y=1|x) = \frac{1}{1+\exp {(-h_{\omega }(x)})} = \sigma \left( h_{\omega }(x) \right) , \end{aligned}$$

where $\sigma$ is the logistic function and $\omega \in \mathbb {R}^n$ is the model parameter that needs to be estimated and *h* is a function that will be specified later. The log-likelihood function of logistic regression for *N* observation pairs $(x^{(1)},y^{(1)}), \dots , (x^{(N)},y^{(N)})$ is

3 $$\begin{aligned} l(\omega |x,y) = \frac{1}{N} \sum _{i=1}^N y^{(i)} \log p_{\omega }^{(i)} + (1-y^{(i)}) \log (1-p_{\omega }^{(i)}), \end{aligned}$$

where $p_{\omega }^{(i)} = p(x^{(i)}|\omega )$. It is the negative of the cross entropy loss. The training model seeks a statistical estimator $\hat{\omega }$ that maximizes this function.

### Optimal Model

The framework described thus far incorporates the tropical, classical and BHV logistic regression as special cases. In this section, we show that these can be distinguished through the choice of the function *h*. In fact, this function *h* can be derived from the conditional distributions *X*|*Y*, as stated in Eq. (4) of Lemma 1, below, by simple application of the Bayes’ rule.

If *X*|*Y* is a Gaussian distribution with appropriate parameters, the resulting model is the classical logistic regression. Alternatively, if *X*|*Y* is a “tropical” distribution, then the resulting classification model is the “tropical” logistic regression. Examples 1 and 2 illustrate this for non-tropical and tropical distributions respectively, and Remark 2 discusses the choice of tropical distribution in more detail.

Furthermore, the function *h* from (4) also minimizes the expected cross-entropy loss according to Proposition 2. Therefore, the *best model* to fit data that have been generated by tropical Laplace distribution (6) is the tropical logistic regression. We conclude this section showing how the tropical metric and tropical Laplace distribution may be applied to produce two intuitive variants of tropical logistic regression, our one- and two-species models.

#### Lemma 1

Let $Y \sim \textrm{Bernoulli}(r)$ and define the random vector $X \in \mathbb {R}^n$ with conditional distribution $X|Y \sim f_Y$, where $f_0, f_1$ are probability density functions defined in $\mathbb {R}^n$. Then, $Y | X \sim \textrm{Bernoulli}(p(X))$ with $p(x) = \sigma (h(x))$, where

4 $$\begin{aligned} h(x) = \log \left( \frac{r f_1(x)}{(1-r)f_0(x)} \right) . \end{aligned}$$

#### Proposition 2

Let $Y \sim \text {Bernoulli}(r)$ and define the random vector $X \in \mathbb {R}^n$ with conditional distribution $X|Y \sim f_Y$, where $f_0, f_1$ are probability density functions defined in $\mathbb {R}^n$. The functional *p* that maximises the expected log-likelihood as given by equation (3) is $p(x) = \sigma (h(x))$, with *h* defined as in equation (4) of Lemma 1.

#### Example 1

(*Normal distribution and classical logistic regression*) Suppose that the two classes are equiprobable ($r=1/2$) and that the covariate is multivariate normal

$$\begin{aligned} X|Y \sim \mathcal {N}(\omega _Y,\sigma ^2 I_n), \end{aligned}$$

where *n* is covariate dimension and $I_n$ is the identity matrix. Using Lemma 1, the optimal model has

5 $$\begin{aligned} h(x) = - \frac{\Vert x-\omega _1\Vert ^2}{2\sigma ^2} + \frac{ \Vert x-\omega _0\Vert ^2}{2\sigma ^2} = \frac{(\omega _1-\omega _0)^T}{\sigma ^2} (x-\bar{\omega }), \end{aligned}$$

where $\Vert \cdot \Vert$ is the Euclidean norm and $\bar{\omega }=(\omega _0+\omega _1)/2$. This model is the *c*lassical logistic regression model with translated covariate $X-\bar{\omega }$ and $\omega = \sigma ^{-2} (\omega _1- \omega _0)$.

#### Example 2

(*Tropical Laplace distribution*) It may be assumed that the covariates are distributed according to the tropical version of the Laplace distribution, as presented in Yoshida et al. (2022b), with mean $\omega _Y$ and probability density functions

6 $$\begin{aligned} f_Y(x) = \frac{1}{\Lambda } \exp \left( - \frac{d_\textrm{tr}(x,\omega _Y)}{\sigma _Y} \right) , \end{aligned}$$

where $\Lambda$ is the normalizing constant of the distribution.

#### Proposition 3

In distribution (6), the normalizing factor is $\Lambda = e! \sigma _Y^{e-1}$.

#### Proof

See “Appendix A”. $$\square $$

#### Remark 2

Consider $\mu \in \mathbb {R}^d$ and a covariance matrix $\Sigma \in \mathbb {R}^{d \times d}$. Then the pdf of a classical Gaussian distribution is

7 $$\begin{aligned} f_{\mu , \Sigma }(x) \propto \exp \left( -\frac{1}{2}(x-\mu )^t \Sigma ^{-1}(x-\mu )\right) \end{aligned}$$

where $x \in \mathbb {R}^d$ and $y^t$ is the transpose of a vector $y \in \mathbb {R}^d$. When $\sigma _Y = 1$, the tropical Laplacian distribution in (6) is tropicalization of the left hand side in (7) where $\Sigma$ is to the tropical identity matrix

$$\begin{aligned} \left( \begin{array}{ccccc} 0 &{}-\infty &{}-\infty &{} \ldots &{}-\infty \\ -\infty &{} 0 &{}-\infty &{} \ldots &{}-\infty \\ \vdots &{} \vdots &{} \vdots &{} \vdots &{}\vdots \\ -\infty &{} -\infty &{}-\infty &{} \ldots &{}0 \\ \end{array} \right) . \end{aligned}$$

Tran (2020) nicely surveys the many different definitions of tropical Gaussian distributions. Since the space of ultrametrics is a tropical linear space (Speyer and Sturmfels 2009), it is natural to use tropical “linear algebra” for the definition of tropical “Gaussian” distribution defined in (6) in this research. Clearly not all desirable properties of the classical Gaussian distribution are necessarily realised in a tropical space.

For example, as Tran discussed in Tran (2020), we lose some natural intuition of orthogonality of vectors. This means that we lose a nice geometric intuition of a correlation between two random vectors. Even with the loss of some nice properties of the classical Gaussian distribution, the tropical Laplacian (7) is a popular choice. It has been applied to statistical analysis of phylogenetic trees: as a kernel density estimator of phylogenetic trees over the space of phylogenetic trees (Yoshida et al. 2022a), and as the Bayes estimator (Huggins et al. 2011) because this distribution is interpretable in terms of phylogenetic trees.

In particular, the tropical metric $d_\textrm{tr}$ represents the biggest difference of divergences (speciation time and mutation rates) between two species among two trees shown in Example 3. This is a very natural and desirable interpretation in terms of phylogenomics. The smaller difference of divergences between two species among the tree with an observed ultrametric *x* and the tree with the centriod has higher probability. Therefore, it is natural to apply a sample generated from the multi-species coalescent model where the species tree has the centroid as its dissimilarity map. It is worth noting that we do not know much about a well-defined distribution over the space of phylogenetic trees, despite many researchers’ attempts (Garba et al. 2021).

**Fig. 1:**
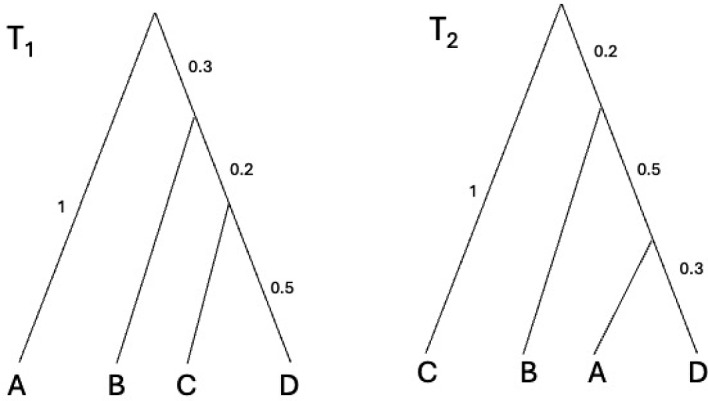
Example for an interpretation of the tropical metric $d_\textrm{tr}$ in Example 3

#### Example 3

(*Tropical Metric*) Suppose we have equidistant trees $T_1$ and $T_2$ with leaf labels $\{A, B, C, D\}$ shown in Fig. 1. Note that leaves *A* and *C* in $T_1$ and $T_2$ are switched. Thus, the pairwise distances from *A* and *D* in $T_1$ and $T_2$, as well as he pairwise distances from *C* and *D* in $T_1$ and $T_2$ are the largest and second largest differences among all possible pairwise distances.

Let *u* be a dissimilarity may from $T_1$ and *v* be a dissimilarity map from $T_2$:

$$\begin{aligned} \begin{array}{ccc} u &{}=&{}(2, 2, 2, 1.4, 1.4, 1) \\ v&{}=&{}(1.6,2,0.6,2,1.6,2). \end{array} \end{aligned}$$

Then we have

$$\begin{aligned} u - v = (2-1.6, 2-2, 2-0.6, 1.4-2, 1.4-1, 1-2) = (0.4, 0,1.4,-0.6,0.4,-1). \end{aligned}$$

Therefore

$$\begin{aligned} d_\textrm{tr}(u, v) = (u - v)_{A, D} - (u - v)_{C, D} \end{aligned}$$

which means the tropical metric measures the difference of divergence between *A* and *D* and difference of divergence between *C* and *D*.

Combining the result of Proposition 3 with Eqs. (4) and (6) yields

8 $$\begin{aligned} h_{\omega _0,\omega _1}(x) = \frac{d_\textrm{tr}(x,\omega _0)}{\sigma _0} - \frac{d_\textrm{tr}(x,\omega _1)}{\sigma _1} + (e-1) \log \left( \frac{\sigma _0}{\sigma _1}\right) . \end{aligned}$$

In its most general form, the model parameters are $(\omega _0,\omega _1,\sigma _0,\sigma _1)$ so the parameter space is a subset of $(\mathbb {R}^e/\mathbb {R}{} \textbf{1})^2 \times \mathbb {R}^2_+$ with dimension 2*e*. Two instances of this general model are particularly practically useful and interpretable. We call these the one-species and two-species models and they will be our focus for tropical logistic regression in the rest of the paper.

**Fig. 2:**
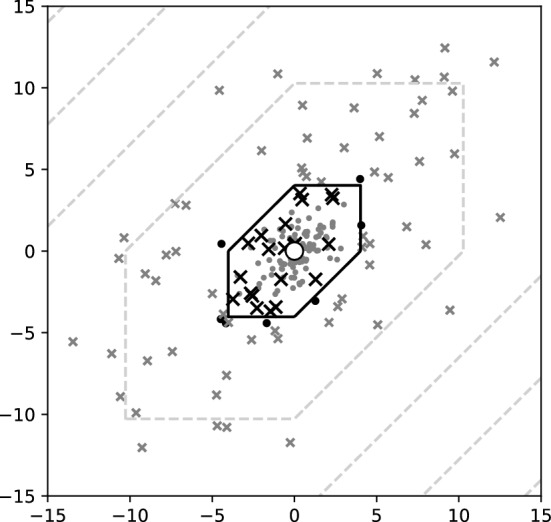
Scatterplot of 200 points—100 dots for class 0 and 100 Xs for class 1, black for misclassified and grey otherwise—imposed upon a contour plot of the probability of inclusion in class 0, where the black contour is the classification threshold. The deviation parameters used in data generation were $\sigma _0=1,\sigma _1=5$ and the centre of the distribution (white-filled point) is the origin. The centres of the two distributions are $\omega _0 = \omega _1$

For the *one-species model*, it is assumed that $\omega _0 =\omega _1$ and $\sigma _0 \ne \sigma _1$. If, without loss of generality, $\sigma _1>\sigma _0$, Eq. (8) becomes

9 $$\begin{aligned} h_{\omega }(x) = \lambda \left( d_\textrm{tr}(x,\omega ) - c \right) , \end{aligned}$$

where $\lambda = (\sigma _0^{-1} - \sigma _1^{-1})$ and $\lambda c = \log {\left( \sigma _1/\sigma _0\right) }$. Symbolically, the expression in Eq. (9) can be considered to be a scaled tropical inner product, whose direct analogue in classical logistic regression is the classical inner product $h_{\omega }(x) = \omega ^T x$. See Section C in the “Appendix” for more details. The classifier is $C(x) = \mathbb {I}(d_\textrm{tr}(x,\hat{\omega } ) > c)$, where $\hat{\omega }$ is the inferred estimator of $\omega ^*$. Note that the classification threshold and the probability contours (*p*(*x*)) are tropical circles, illustrated in Fig. 2.

For the *two-species-tree model*, it is assumed that $\sigma _0 = \sigma _1$, and $\omega _0\ne \omega _1$. Equation (8) reduces to

10 $$\begin{aligned} h_{\omega _0,\omega _1}(x) = \sigma ^{-1}( d_\textrm{tr}(x,\omega _0) - d_\textrm{tr}(x,\omega _1) ), \end{aligned}$$

with a classifier $C(x) = \mathbb {I}( d_\textrm{tr}(x,\hat{\omega }_0) > d_\textrm{tr}(x,\hat{\omega }_1) ),$ where $\hat{\omega }_y$ is the inferred tree for class $y \in \{0,1\}$. The classification boundary is the tropical bisector which is extensively studied in Criado et al. (2021) between the estimators $\hat{\omega }_0$ and $\hat{\omega }_1$ and the probability contours are tropical hyperbolae with $\hat{\omega }_0$ and $\hat{\omega }_1$ as foci, as shown in Fig. 4(right).

The one-species model is appropriate when the gene trees of both classes are concentrated around the same species tree $\omega$ with potentially different concentration rates. When the gene trees of each class come from distributions centered at different species trees the two-species model is preferred.

### Model selection

In the previous subsection, we established the correspondence between the covariate conditional distribution and the function *h* which defines the logistic regression model. According to Proposition 2, the best regression model follows from the distribution that fits the data. The family of distributions that best fits the training data of a given class can indicate which regression model to use. The question that naturally arises is how to assess which family of conditional distributions has the best fit.

One issue is that the random covariates are multivariate and so the Kolmogorov-Smirnov test can not be readily applied. Moreover, the four families considered, namely the classical and tropical Laplace and Gaussian distributions, are not nested. Nonetheless, it is observed that for all these families the distances of the covariates from their centres are Gamma distributed. This is stated in Corollary 1 which is based on Proposition 4. Note that the distance metric corresponds to the geometry of the covariates. However, the arguments used in the proof of Corollary 1 do not work for distributions defined on the space of ultrametric trees $\mathcal {U}_m$, because these spaces are not translation invariant. For a similar reason, the corollary does not apply to the BHV metric.

#### Proposition 4

Consider a function $d:\mathbb {R}^n \rightarrow \mathbb {R}$ with $\alpha d(x) = d(\alpha x)$, for all $\alpha \ge 0$. If $X \sim f$ with $f(x) \propto \exp (-d^i(x)/(i\sigma ^i))$ being a valid probability density function, for some $i \in \mathbb {N},$ $\sigma >0$. Then, $d^i(X) \sim i \sigma ^i \textrm{Gamma}(n/i)$.

#### Corollary 1

If $X \in \mathbb {R}^e$ with $X\sim f \propto \exp {(-d^i(x,\omega ^*)/(i\sigma ^{i}))}$, where *d* is the Euclidean metric, then $d^i(X,\omega ^*) \sim i \sigma ^i \textrm{Gamma}(e/i)$. If $X \in \mathbb {R}^e/\mathbb {R}{} \textbf{1}$ with $X\sim f \propto \exp {(-d_\textrm{tr}^i(x,\omega ^*)/(i\sigma ^{i}))}$, where $d_\textrm{tr}$ is the tropical metric, then $d_\textrm{tr}^i(X,\omega ^*) \sim i \sigma ^i \textrm{Gamma}((e-1)/i)$.

The suitability of the tropical against the classical logistic regression is assessed for the coalescent model and the Mr Bayes trees, by visually comparing the fits of the theoretical Gamma distributions to Euclidean and tropical distances of the gene trees to the species tree.

### Consistency and Generalization Error

In this subsection, the consistency of the statistical estimators (in Theorem 2) and of the tropical logistic regression as a learning algorithm (in Propositions 5 and 6) are established. Finally, the generalization error (probability of misclassification for unseen data) for the one-species model is derived and an upper bound is found for the generalization error of the two-species model. In both cases the error bounds are getting better as the estimation error $\epsilon$ shrinks to zero. It is worth mentioning that in the case of exact estimation, the generalization error of the one-species model can be computed explicitly by Eq. (11). Moreover, there is a higher misclassification rate from the more dispersed class (inequality (12)).

#### Theorem 2

(Consistency) The estimator $(\hat{\omega },\hat{\sigma }) = (\hat{\omega }_0, \hat{\omega }_{1}, \hat{\sigma }_0,\hat{\sigma }_1) \in \Omega ^2 \times \Sigma ^2$ of the parameter $(\omega ^*,\sigma ^*) = (\omega _0^*,\omega _1^*,\sigma _0^*,\sigma _1^*) \in \Omega ^2 \times \Sigma ^2$ is defined as the maximizer of the logistic likelihood function, where $\Omega \subset \mathbb {R}^e/\mathbb {R}{} \textbf{1}$ and $\Sigma \subset \mathbb {R}_+$ are compact sets. Moreover, it is assumed that the covariate-response pairs $(X_1,Y_1), (X_2,Y_2), \dots , (X_n,Y_n)$ are independent and identically distributed with $X_i \in \mathbb {R}^e/\mathbb {R}{} \textbf{1}$, $d_\textrm{tr}(X,\omega _Y)$ being integrable and square-integrable and $Y_i \sim \text {Bernoulli}( S(h(X_i,(\omega ^*,\sigma ^*) )))$. Then,

$$\begin{aligned} (\hat{\omega },\hat{\sigma }) \overset{p}{\rightarrow }\ (\omega ^*,\sigma ^*) \text { as } n \rightarrow \infty . \end{aligned}$$

In other words, the model parameter estimator is consistent.

#### Proposition 5

(One-species generalization error) Consider the one-species model where $\omega =\omega _0=\omega _1 \in \mathbb {R}^e/\mathbb {R}{} \textbf{1}$ and without loss of generality $\sigma _0 < \sigma _1$. The classifier is $C(x)=\mathbb {I}(h_{\hat{\omega }}(x) \ge 0)$, where *h* is defined in equation (9) and $\hat{\omega }$ is the estimate for $\omega ^{\star }$. Define the covariate-response joint random variable (*X*, *Y*) with $Z = \sigma _Y^{-1} d_\textrm{tr}(X,\omega _Y^*)$ drawn from the same distribution with cumulative density function *F*. Then,

$$\begin{aligned} \mathbb {P}(C(X) = 1|Y=0)&\in \left[ 1-F(\sigma _1 \left( \alpha + \epsilon \right) ), 1-F(\sigma _1 \left( \alpha - \epsilon \right) ) \right] , \\ \mathbb {P}(C(X) = 0|Y=1)&\in \left[ F(\sigma _0 \left( \alpha - \epsilon \right) ), F(\sigma _0 \left( \alpha + \epsilon \right) ) \right] , \text { where } \\ \alpha = \frac{\log {\frac{\sigma _1}{\sigma _0}}}{\sigma _1 - \sigma _0},&\,\, \text { and } \,\, \epsilon = (e-1) \frac{d_\textrm{tr}( \hat{\omega }, \omega ^* ) }{\sigma _1\sigma _0}. \end{aligned}$$

The generalization error defined as $\mathbb {P}(C(X) \ne Y)$ lies in the average of the two intervals above. In particular, note that if $\hat{\omega } = \omega ^*$, then $\epsilon =0$ and the intervals shrink to a single point, so the misclassification probabilities and generalization error can be computed explicitly.

11 $$\begin{aligned} \mathbb {P}(C(X) \ne Y) = \frac{1}{2} \left( 1-F(\sigma _1 \alpha ) + F(\sigma _0 \alpha ) \right) \end{aligned}$$

Moreover, if $\hat{\omega } = \omega _*$ and $Z \sim \textrm{Gamma}(e-1,1)$, then

12 $$\begin{aligned} \mathbb {P}(C(X)=1 | Y = 0) < \mathbb {P}(C(X)=0 | Y = 1). \end{aligned}$$

#### Proposition 6

(Two-species generalization error) Consider the random vector $X \in \mathbb {R}^e/\mathbb {R}{} \textbf{1}$ with response $Y \in \{0,1\}$ and the random variable $Z = d_\textrm{tr}( X, \omega ^*_Y )$. Assuming that the probability density function is $f_X(x) \propto f_Z(d_\textrm{tr}( x, \omega ^*_Y ))$, the generalization error satisfies the following upper bound

13 $$\begin{aligned} \mathbb {P}\left( C(X) \ne Y \right) \le \frac{1}{2} F^C_{Z}(\Delta _{\epsilon }) + h(\epsilon ) , \end{aligned}$$

where $\epsilon = d_\textrm{tr}(\hat{\omega }_1, \omega ^*_1 )+d_\textrm{tr}(\hat{\omega }_{0}, \omega ^*_{0})$, $2\Delta _\epsilon = \left( d_\textrm{tr}(\omega _1^*, \omega _{0}^*) - \epsilon \right)$, $F^C_Z$ is the complementary cumulative distribution of *Z*, and $h(\epsilon )$ is an increasing function of $\epsilon$ with $2\,h(\epsilon ) \le F^C_Z(\Delta _{\epsilon })$ and $h(0)=0$ assuming that $\mathbb {P}(d_\textrm{tr}(X,\omega _1^*)) = d_\textrm{tr}(X,\omega _{-1}^*) )=0$.

Moreover, under the conditions of Theorem 2, our proposed learning algorithm is consistent.

Observe that the upper bound is a strictly increasing function of $\epsilon$.

#### Example 4

The complementary cumulative distribution of $\textrm{Gamma}(n,\sigma )$ is $F^C(x) = \Gamma (n,x/\sigma )/\Gamma (n,0)$, where $\Gamma$ is the upper incomplete gamma function and $\Gamma (n,0)=\Gamma (n)$ is the regular Gamma function. Therefore, the tropical distribution given in Eq. (6) yields the following upper bound for the generalization error

14 $$\begin{aligned} \frac{\Gamma \left( e-1, \frac{d_\textrm{tr}(\omega _0^*,\omega _1^*)}{2\sigma } \right) }{2\Gamma (e-1)}, \end{aligned}$$

under the assumptions of Proposition 6 and assuming that the estimators coincide with the theoretical parameters. This assumption is reasonable for large sample sizes and it follows from Theorem 2.

In subsequent sections, these theoretical results will guide us in implementing our model. Bounds on the generalization error from Propositions 5 and 6 are computed and the suitability of Euclidean and tropical distributions, and as a result of classical and tropical logistic regards, is assessed using the distance distribution of Proposition 4.

## Optimization

As in the classical logistic regression, the parameter vectors $({\hat{\omega }}, {\hat{\sigma }})$ maximising the log-likelihood (3), are chosen as statistical estimators. Identifying these requires the implementation of a continuous optimization routine. While root-finding algorithms typically work well for identifying maximum likelihood estimators in the classical logistic regression where the log-likelihood is concave, they are unsuitable here. The gradients of the log-likelihood under the proposed tropical logistic models are only piecewise continuous, with the number of discontinuities increasing along with the sample size. Furthermore, even if a parameter is found, it may merely be a local optimum. In light of this, the tropical Fermat–Weber problem of Lin and Yoshida (2018) is revisited.

### Fermat–Weber Point

A Fermat–Weber point or geometric mean $\tilde{\omega }_n$ of the sample set $(X_1,\dots ,X_n)$ is a point that minimizes the sum of distances from to sample points, i.e.

15 $$\begin{aligned} \tilde{\omega }_n \in \mathop {{{\,\mathrm{arg\,min}\,}}}\limits _{\omega } \sum _{i=1}^n d_{\textrm{tr}}(X_i,\omega ). \end{aligned}$$

This point is rarely unique for finite *n*, indeed there will often be an infinite set of Fermat–Weber points Lin and Yoshida (2018). However, the proposition below gives conditions for asymptotic convergence.

#### Proposition 7

Let $X_i \overset{\textrm{iid}}{\sim }\ f$, where where *f* is a distribution that is symmetric around its center $\omega ^* \in \mathbb {R}^{e}/\mathbb {R}{} \textbf{1}$ i.e. $f(\omega ^* + \delta ) = f(\omega ^* - \delta )$ for all $\delta \in \mathbb {R}^{e}/\mathbb {R}{} \textbf{1}$. Let $\tilde{\omega }_n$ be any Fermat–Weber point as defined in Eq. (15). Then, $\tilde{\omega }_n \overset{p}{\rightarrow }\ \omega ^*$ as $n \rightarrow \infty$.

The significance of Proposition 7 is twofold. It proves that the Fermat–Weber sets of points sampled from symmetric distributions tend to a unique point. This is a novel result and ensures that for sufficiently large sample sizes the topology of any Fermat–Weber point is fixed. Additionally, using Theorem 2 and Proposition 7, $\hat{\omega }_n - \tilde{\omega }_n \overset{p}{\rightarrow }\ 0$ as $n \rightarrow \infty$. Furthermore, empirical evidence in Fig. 5, see the following section, suggests that $d_\textrm{tr}(\hat{\omega }_n, \omega ^*) = \mathcal {O}_p(1/\sqrt{n})$ and $d_\textrm{tr}(\tilde{\omega }_n, \omega ^*) = \mathcal {O}_p(1/\sqrt{n})$. These statements are left as conjectures and proofs of them are beyond the scope of this paper. Assuming they hold and applying triangular inequality, it follows that $d_\textrm{tr}(\hat{\omega }_n, \tilde{\omega }_n)= \mathcal {O}_p(1/\sqrt{n}).$ As a result, for a sufficiently large sample size we may use the Fermat–Weber point as an approximation for the MLE vector. Indeed, there are benefits in doing so.

Instead of having a single optimization problem with $2e-1$ variables, three simpler problems are considered; finding the Fermat–Weber point of each of the two classes, which has $e-1$ degrees of freedom and then finding the optimal $\sigma$ which is a one dimensional root finding problem. The algorithms of our implementation for both model can be found in “Appendix D”.

There is also another another benefit of using Fermat–Weber points. Proposition 8 provides a sufficient optimality condition that the MLE lacks, since a vanishing gradient in the log likelihood function merely shows that there is a local optimum.

#### Proposition 8

Let $X_1,\dots ,X_n \in \mathbb {R}^e/\mathbb {R}{} \textbf{1}$, $\omega \in \mathbb {R}^e/\mathbb {R}{} \textbf{1}$ and define the function

$$\begin{aligned} f(\omega ) = \sum _{i=1}^{n} d_\textrm{tr}(X_i,\omega ). \end{aligned}$$

i. The gradient vector of *f* is defined at $\omega$ if and only if the vectors $\omega - X_i$ have unique maximum and minimum components for all $i \in [n]$.
ii. If the gradient of *f* at $\omega$ is well-defined and zero, then $\omega$ is a Fermat–Weber point.

In Lin and Yoshida (2018), Fermat–Weber points are computed by means of linear programming, which is computationally expensive. Employing a gradient-based method is much faster, but there is no guarantee of convergence. Nevertheless, if the gradient, which is an integer vector, vanishes, then it is guaranteed, as above, that the algorithm has reached a Fermat–Weber point. This tends to happen rather frequently, but not in all cases examined in Sect. 4.

#### Remark 3

Our choice of Fermat–Weber points to represent centers is not the only practical option, however it is an especially desirable choice due to the interpretability of its resulting solutions.

Recently, Comǎneci and Joswig studied tropical Fermat–Weber points obtained using the asymmetric tropical distance (Comǎneci and Joswig 2023). They found that if all $X_i$ are ultrametric, then the resulting tropical Fermat–Weber points are also ultrametric, all with the same tree topology. On the other hand, Lin et al. (2017) show that a tropical Fermat–Weber point defined with $d_\textrm{tr}$ of a sample taken from the space of ultrametrics could fall outside of the ultrametric space.

Despite this, the major drawback of using the asymmetric tropical distance, is that it would result in losing the phylogenetic interpretation of the distance or dissimilarity between two trees held by the tropical metric $d_\textrm{tr}$—see Remark 2.

## Results

In this section, tropical logistic regression is applied in three different scenarios. The first and simplest considers datapoints generated from the tropical Laplace distribution. Secondly, gene trees sampled from a coalescent model are classified based on the species tree they have been generated from, and finally it is applied as an MCMC convergence criterion for the phylogenetic tree construction, using output from the Mr Bayes software. The models’ performance in terms of misclassification rates and AUCs on these datasets is examined.

### Toy Example

In this example, a set of data points is generated from the tropical normal distribution as defined in Eq. (6) using rejection sampling.

The data points are defined in the tropical projective torus $\mathbb {R}^e/\mathbb {R}{} \textbf{1}$, which is isomorphic to $\mathbb {R}^{e-1}$. To map $x \in \mathbb {R}^e/\mathbb {R}{} \textbf{1}$ to $\mathbb {R}^{e-1}$, simply set the last component of *x* to 0, or in other words $x \mapsto (x_1-x_e,x_2-x_e, \dots , x_{e-1} - x_e)$. For illustration purposes, it is desirable to plot points in $\mathbb {R}^2$, so we use $e=3$ which corresponds to phylogenetic trees with 3 leaves. Both the one-species model and the two-species model are examined.

**Fig. 3:**
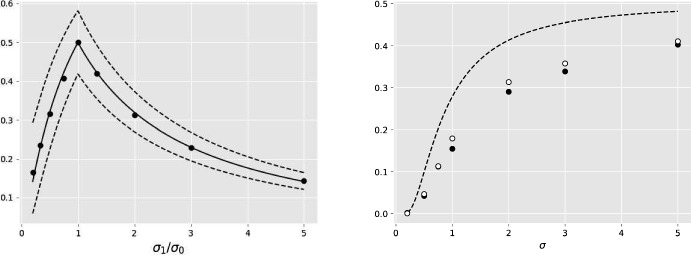
(left) Generalization error for 9 different deviation ratios. The estimator $\hat{\omega } = (0.3,0,3)$ differs from the true parameter $\omega = (0,0)$. The upper and lower bounds of Proposition 5 are plotted in dashed lines and the generalization error for the correct estimator $\hat{\omega }= \omega ^*$ plotted in solid line. The dots represent the proportion of misclassified points from a set of 2000 points in each experiment, 1000 points for each class. (right) Generalization errors for 7 different dispersion parameters with black markers for the two-species tropical logistic regression and white markers for the classical logistic regression. The upper bound (14) of Proposition 6 is plotted in dashed line

In the case of the former, $\omega = \omega _0 = \omega _1$ and $\sigma _0 \ne \sigma _1$. The classification boundary in this case is a tropical circle. If $\sigma _0 < \sigma _1$, the algorithm classifies points close to the inferred centre to class 0 and those that are more dispersed away from the centre as class 1. For simplicity, the centre is set to be the origin $\omega =(0,0,0)$ and no inference is performed. In Fig. 2 a scatterplot of of the two classes is shown, where misclassified points are highlighted. As anticipated from Proposition 5 there are more misclassified points from the more dispersed class (class 1). Out of 100 points for each class, there are 7 and 21 misclassified points from class 0 and 1 respectively, while the theoretical probabilities calculated from Eq. (11) of Proposition 5 are $9\%$ and $19\%$ respectively.

**Fig. 4:**
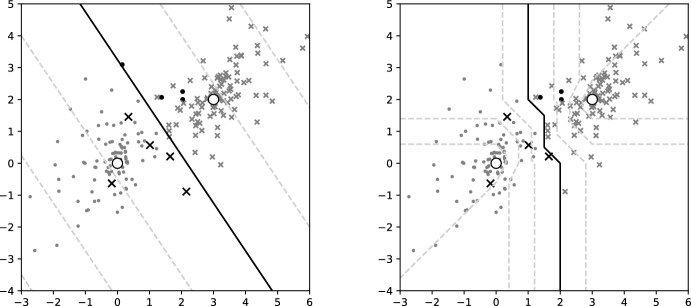
Scatterplot of points—dots for class 0 and X for class 1, black for misclassified according to (left) *classical logistic regression* or (right) *tropical logistic regression*, and grey otherwise—alongside a contour plot of the probabilities, where the black contour is the classification threshold. The centres, drawn as big white dots, are $\omega _0 = (0,0,0), \omega _1=(3,2,0)$ and $\sigma = 0.5$

Varying the deviation ratio $\sigma _1/\sigma _0$ in the data generation process allows exploration of its effect on the generalization error in the one-species model. The closer this ratio is to unity, the higher the generalization error. For $\sigma _0 = \sigma _1$ the classes are indistinguishable and hence any model is as good as a random guess i.e. the generalization error is 1/2. The estimate of the generalization error for every value of that ratio is the proportion of misclassified points in both classes. Assuming an inferred $\omega$ that differs from the true parameter, Fig. 3(left) verifies the bounds of Proposition 5.

For the two-species model, tropical logistic regression is directly compared to classical logistic regression. Data is generated using different centres $\omega _0 = (0,0,0),$ $\omega _1 = (3,2,0)$ but the same $\sigma =0.5$. The classifier is $C(x)=\mathbb {I}(h(x)>0)$ for both methods, using *h* as defined in Eqs. (5) and (10) for the classical and tropical logistic regression respectively. Figure 4 compares contours and classification thresholds of the classical (left) and tropical (right) logistic regression by overlaying them on top of the same data. Out of $100+100$ points there are $5+4$ and $4+3$ misclassifications in classical and tropical logistic regression respectively. Figure 3(right) visualizes the misclassification rates of the two logistic regression methods for different values of dispersion $\sigma$, showing the tropical logistic regression to have consistently lower generalization error than the classical, even in this simple toy problem.

**Fig. 5:**
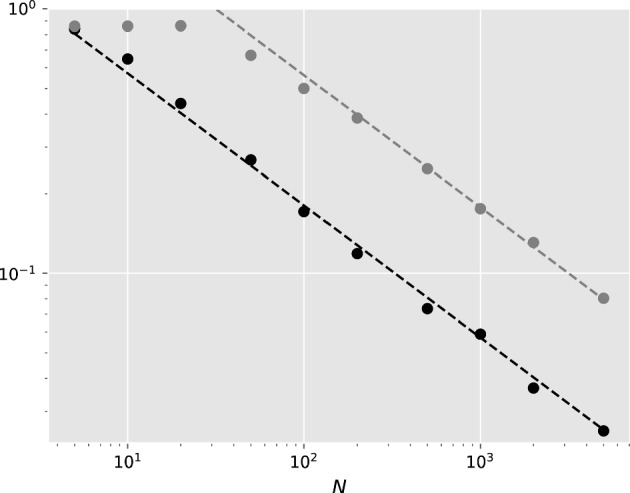
Expected asymptotic error for FW points $(\tilde{\omega }_0)_N$ (in black) and MLE points $(\hat{\omega }_0)_N$ (in grey) for different values of *N*. Error is defined as the tropical distance from the true centre $\omega _0^*$ i.e. $d_\textrm{tr}(\omega _N,\omega _0^*)$. The dashed lines are $y \propto N^{-0.5}$, so this figure illustrates that $d_\textrm{tr}((\omega _0)_N,\omega _0^*) = \mathcal {O}_p(1/\sqrt{N})$ as $N\rightarrow \infty$

Finally, we investigate the convergence rate of the Fermat–Weber points and of the MLEs from the two-species model as the sample size *N* increases. Fixing $\omega _0^* = (0,0,0)$ and $\omega _1^* = (3,2,0)$ as before, the Fermat–Weber point numerical solver and the log-likelihood optimization solver are employed to find $(\tilde{\omega }_0)_N$ and $( (\hat{\omega }_0)_N, (\hat{\omega }_1)_N, \hat{\lambda }_N )$ respectively. From this, the error is computed for the two methods, which is defined as $d_N = d_\textrm{tr}((\omega _0)_N,\omega _0^*)$ for $(\omega _0)_N= (\tilde{\omega }_0)_N$ and $(\hat{\omega }_0)_N$ respectively. For each *N*, we repeat this procedure 100 times to get an estimate of the mean error rate $r_N = \mathbb {E}\left( d_N \right)$. Figure 5 shows that for both methods, $r_N \sqrt{N} \rightarrow C$ as $N \rightarrow \infty$, with $C_\textrm{FW} < C_\textrm{MLE}$. Since $\mathbb {E}(\sqrt{N} d_N) \rightarrow C$, it follows that $\sqrt{N} d_N = \mathcal {O}_p(1)$ as $N \rightarrow \infty$. This supports the assumption of Sect. 3 that Fermat–Weber points can be used in lieu of MLEs, since they converge to each other in probability at rate $1/\sqrt{N}$. Interestingly, the MLEs produce higher errors than FW points. This may be due to an imperfection of the MLE solver, which may be stuck at a local optimum.

### Coalescent Model

The data that have been used in our simulations were generated under the multispecies coalescent model, using the python library dendropy (Sukumaran and Holder 2010). The classification method we propose is the two-species model because two distinct species tree have been used to generate gene tree data for each class.

Two distinct species trees are used, which were randomly generated under a Yule model. Then, using dendropy, 1000 gene trees are randomly generated for each of the two species. The trees have 10 leaves and so the number of the model variables is $$\left( {\begin{array}{c}10\\ 2\end{array}}\right) = 45$$. They are labelled according to the species tree they are generated from. The tree generation is under the coalescent model for specific model parameters.

Since the species trees are known, we conduct a comparative analysis between classical, tropical and a BHV-based (Billera et al. 2001) logistic regression. In the “Appendix”, we show an approximation analog of our model to the BHV metric. The comparative analysis includes the distribution fitting of distances and the misclassification rates for different metrics.

**Fig. 6:**
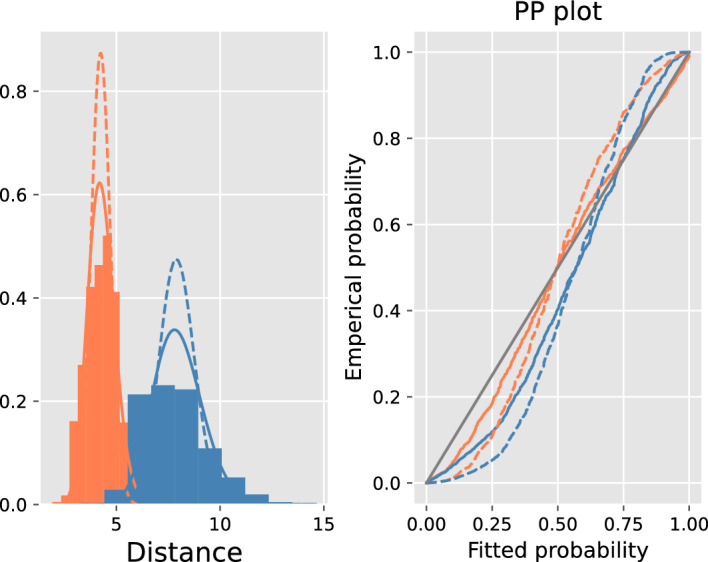
(Left) Histograms of the distances of 1000 gene trees from the species trees that generated them under the coalescent model with $R=0.7$. Coral and blue corresponds to tropical and euclidean geometries respectively. The solid and dashed lines are fitted distributions $\sigma \textrm{Gamma}(n)$ and $\sigma \sqrt{\chi ^2_{n}}$ respectively; $\sigma$ is chosen to be the MLE, derived in the “Appendix”. Euclidean metric has worse fit than the tropical metric. This can also be observed by the corresponding pp-plots (right) (Color figure online)

In Fig. 6, the distribution of the radius $d(X,\omega )$ as given by Proposition 4, is fitted to the histograms of the Euclidean and tropical distances of gene trees to their corresponding species tree, along with the corresponding pp-plots on the right. According to Proposition 4, for both the classical and tropical Laplace distributed covariates, $d(X,\omega ^*) \sim \sigma \textrm{Gamma}(n)$, shown in solid lines in Fig. 6, where $n = e = 45$ and $n=e-1=44$ for the classical and tropical case respectively. Similarly, for normally distributed covariates, $d(X,\omega ^*) \sim \sigma \sqrt{\chi _{n}^2}$, shown in dashed lines. It is clear that Laplacian distributions produce better fits in both geometries and that the tropical Laplacian fits the data best. As discussed in Sect. 2.2, the same analysis can not be applied to the BHV metric, because the condition of Proposition 4 does not hold.

**Fig. 7:**
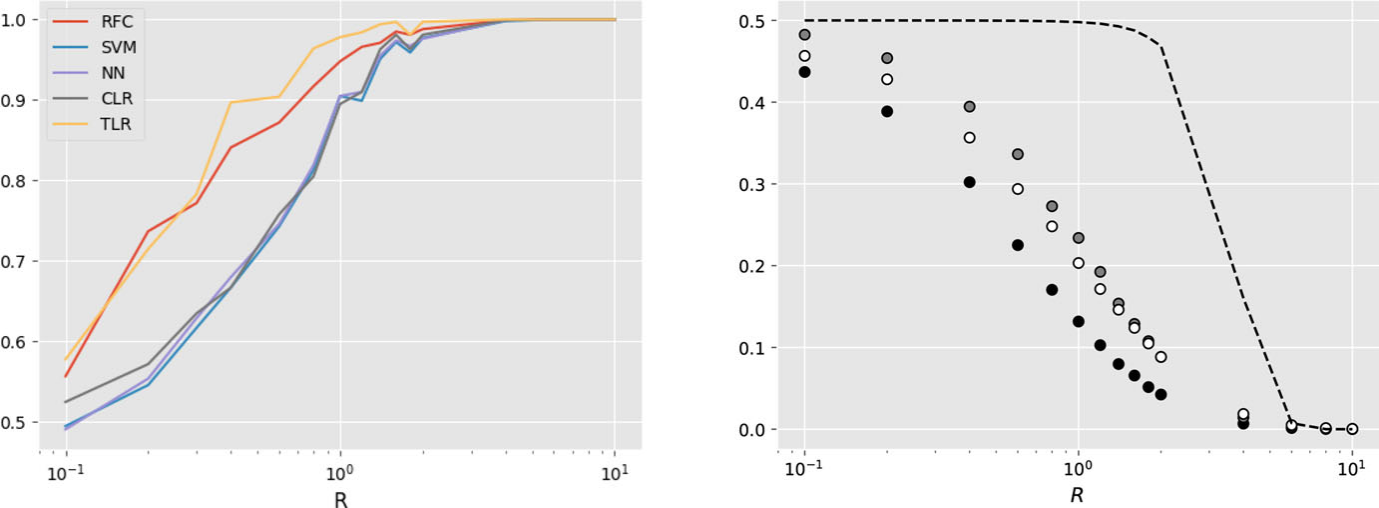
(left) Average AUCs against *R*. Five classification models which we considered are the tropical two species-tree model (TLR), random forest classifier (RFC), support vector machines (SVM), neural networks (NN) and classical logistic regression (CLR). We used default set up for TLR, SVM, NN and CLR implemented by sklearn. (right) the x-axis represents the ratio *R* and the y-axis represents misclassification rates. Black circles represent the tropical logistic regression, white circles represent the classical logistic regression, grey points represent the logistic regression with BHV metric, and the dashed line represents the theoretical generalization error shown in Proposition 6 (color figure online)

*Species depth* $\text {SD}$ is the time since the speciation event between the species and *effective population size* *N* quantifies genetic variation in the species. Datasets have been generated for a range of values $R:= \textrm{SD}/N$ by varying species depth. For low values of *R*, speciation happens very recently and so the gene trees look very much alike. Hence, classification is hard for datasets with low values of *R* and vice versa, because the gene deviation $\sigma _R$ is a decreasing function of *R*. We expect classification to improve in line with *R*. Figures 11 and 12 in “Appendix H” confirm that, by showing that as *R* increases the receiver operating characteristic (ROC) curves are improving and the Robinson–Foulds and tropical distances of inferred (Fermat–Weber point) trees are decreasing. In addition, Fig. 7 shows that as *R* increases, AUCs increase (left) and misclassification rates decrease (right). It also shows that tropical logistic regression produces higher AUCs than classical logistic regression and other out-of-the-box ML classifiers such as random forest classifier, neural networks with a single sigmoid output layer and support vector machines. Our model also produces lower misclassification rates than both the BHV and classical logistic regression. Finally, note that the generalization error upper bound as given in Eq. (14) is satisfied but it not very tight (dashed line in Fig. 7).

### Convergence of Mr Bayes

Mr Bayes (Huelsenbeck et al. 2001) is a widely used software for Bayesian inference of phylogeny using MCMC to sample the target posterior distribution. An important feature of the software is the diagnostic metrics indicating whether a chain has converged to the equilibrium distribution. This is calculated at regular, specified intervals, set by the variable diagnfreq, using the average standard deviation of split frequencies (ASDSF introduced by Lakner et al. (2008)) between two independently run chains. The more similar the split frequencies between the two chains are, the lower the ASDSF, and the more likely it is that both chains have reached the equilibrium distribution.

Our classification model provides an alternative convergence criterion for MCMC convergence. Consider two independently run chains; the sampled trees of the two chains correspond to two classes and the AUC value is a measure of how distinguishable the two chains are. High values of AUC are associated with easily distinguishable chains, implying that the chains have not converged to the equilibrium distribution. At every iteration that is a multiple of diagnfreq, the ASDSF metric is calculated and the AUC of the two chains is found by applying tropical logistic regression to the truncated chains that only keep the last $30 \%$ of the trees in each chain.

**Fig. 8:**
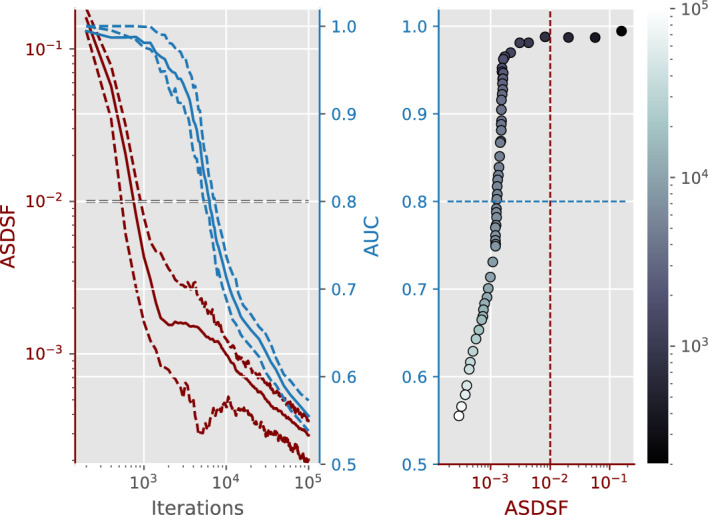
(Left) Average ASDSF (in red) and AUC (in blue) values plotted against the number of iterations of the MCMC chains. The coloured dashed lines correspond to the first and third quartile. The grey dashed line indicates the Mr Bayes threshold for ASDSF and our provisional AUC threshold of $80\%$. (Right) ASDSF and AUC values plotted against each other, with the iterations coloured according to the colourbar and the dashed lines corresponding to the thresholds for each metric (Color figure online)

For our comparison study, the data used were the gene sequences from the primates.nex file. This dataset comes with the Mr Bayes software and it is used as an example in Ronquist et al. (2005). Figure 8 shows the two metrics at different iterations of the two independent chains ran on this dataset. According to the Mr Bayes manual, the convergence threshold for their metric is $10^{-2}$. This is achieved at the 800th iteration, when our method produces an AUC of $97\%$, which indicates that the chains may have not converged yet, contrary to the suggestion of Mr Bayes. A likely explanation for this discrepancy is the dependence of ASDSF on tree topologies instead of branch lengths. The frequencies of the tree topologies may have converged to those of the equilibrium distribution, even if the branch lengths have not. Eventually, the AUC values drop rapidly when iterations exceed $2\times 10^3$, while the ASDSF metric is reduced at a much slower rate. In this second phase, the branch lengths are calibrated, while the topology frequencies do not change a lot. Finally, for iterations that exceed $10^5$, neither metric can reject convergence, with ASDSF being 10 lower than the threshold and the AUC values finally dropping below $70\%$, which is a typical threshold for poor classification.

**Fig. 9:**
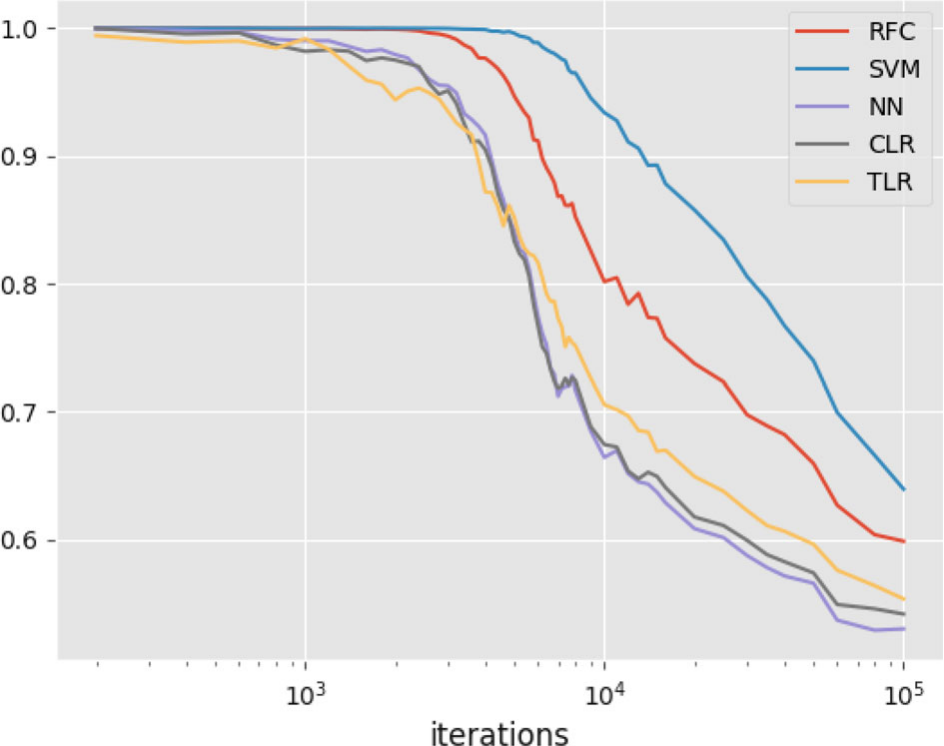
Average AUC values plotted against the number of MCMC iterations for the 5 supervised learning methods considered (color figure online)

**Fig. 11:**
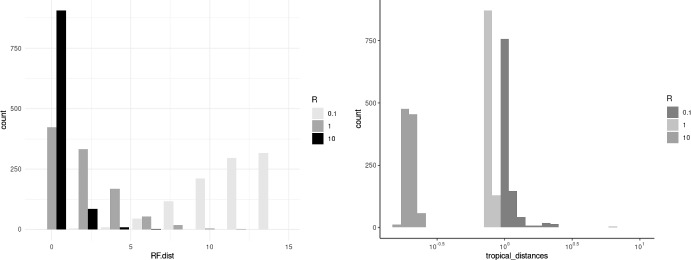
(left) Robinson–Foulds distances and (right) tropical distances of inferred species trees $\hat{\omega }$ from the actual species trees $\omega ^*$ for $R=0.1,1,10$

**Fig. 12:**
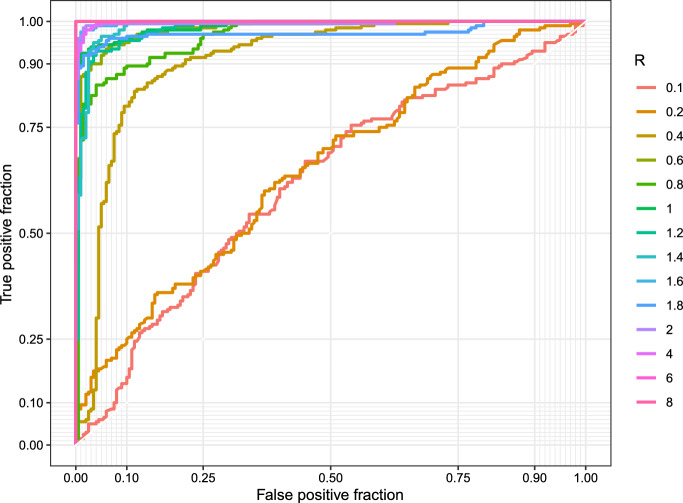
ROC curves for the tropical logistic regression with different values of *R*. Higher the value of *R* is the closer an estimated ROC curve for the tropical logistic regression model gets to the point (0, 1) (color figure online)

When our classification method is compared to other classifiers, it marginally outperforms classical logistic regression and neural networks with a single sigmoid output but underperforms support vector machines and random forest classifiers (Fig. 9). Despite their simplicity, logistic regression models cannot capture the complexity of the chain classification problem. More advanced statistical methods that conform to tropical geometry (such as tropical support vector machines Yoshida et al. 2023a) could be applied instead at the cost of simplicity and interpretability.

## Discussion

In this paper we developed a tropical analog of the classical logistic regression model and considered two special cases; the one species-tree model and two species-tree model. In our empirical work the two-species model was most effective, but we anticipate both are potentially impactful tools for phylogenomic analysis. The one-species model’s principal benefit is having the same number of parameters as the number of predictors, unlike the two-species model which has almost twice as many. Therefore, the one-species model more readily fits the standard definition of a generalized linear model and could generalize to a stack of GLMs to produce a “tropical” neural network, which is investigated in Yoshida et al. (2023b).

The two-species model implemented on data generated under the coalescent model outperformed classical and BHV logistic regression models in terms of misclassification rates, AUCs and fitness of the distribution of distances to their centre. It was also observed that Laplacian distributions were better fitting than Gaussians, for both geometries. Empirically selecting tropical distributions over Euclidean distributions suffices for the scope of this paper, but further theoretical justification of the suitability of such distributions is needed. Moreover, further research on the generalization error for the two-species model would provide tighter bounds.

Finally, the AUC metric of our model is proposed as an alternative to the ASDSF metric for MCMC convergence checking. Our metric is more conservative and robust, taking branch lengths into account. Nonetheless, computing the ASDSF is less computationally intensive than running our method. There seems to be a tradeoff between the reliability of the convergence criterion tool and computational speed. Further research can shed light on the types of datasets where the ASDSF metric becomes unreliable. Then, the two metrics could complement each other, with our methods applied only when there is a good indication that ASDSF is unreliable.

## Figures and Tables

**Algorithm 1:**
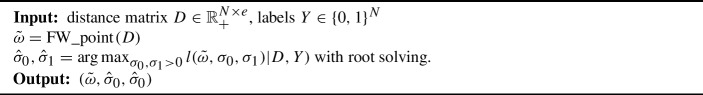
One-species tropical logistic regression

**Algorithm 2:**
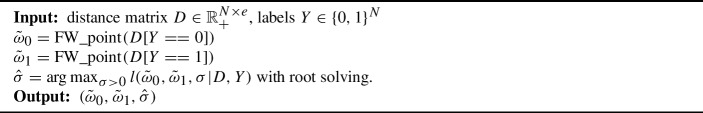
Two-species tropical logistic regression

**Fig. 10:**
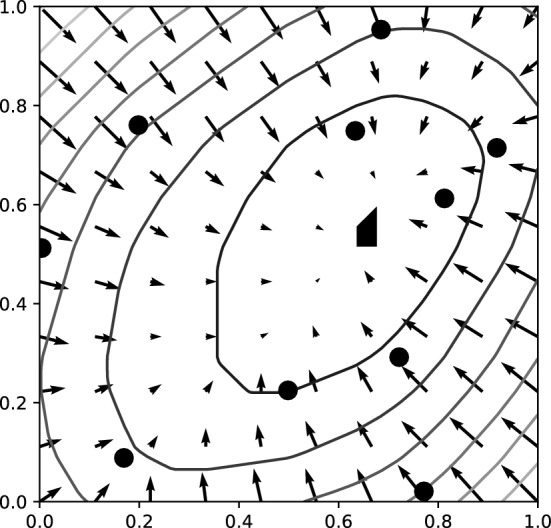
Visualization of the function $f(\omega )= \sum _{i=1}^{10} d_\textrm{tr}(X_i,\omega )$ for $X_i$. The black circles are the datapoints $X_1, \dots , X_{10}$, the solid lines are contours of *f*, the vector field is the gradient and the small black trapezoid at (0.65, 0.55) is the Fermat–Weber set

## Data Availability

DRYAD with DOI: 10.5061/dryad.tht76hf65.

## Appendix A: Proofs

### Proof of Lemma 1

A simple application of the Bayes rule for continuous random variables yields

$$\begin{aligned} p(x) = \mathbb {P}(Y=1|X=x)&= \frac{f_1(x) \mathbb {P}(Y=1)}{f_0(x) \mathbb {P}(Y=0) + f_1(x) \mathbb {P}(Y=1)} \\&= \frac{1}{1 + \frac{f_1(x) (1-r)}{f_0(x) r} } = S(h(x)). \end{aligned}$$

$$\square $$

### Proof of Proposition 2

The expected log-likelihood is expressed as

$$\begin{aligned} \mathbb {E}(l)&= \mathbb {E}\left( Y \log (p(X)) + (1-Y) \log (1-p(X)) \right) \\&= \mathbb {P}(Y=1) \int _{\mathbb {R}^n} f_1(x) \log (p(x)) \, dx \\&\quad + \mathbb {P}(Y=0) \int _{\mathbb {R}^n} f_0(x) \log (1-p(x)) \, dx \\&= \int _{\mathbb {R}^n} L(x,p(x)) \, dx , \end{aligned}$$

where $L(x,p) = r f_1(x) \log (p) + (1-r) f_0(x) \log (1-p)$ is treated as the Lagrangian. The Euler-Lagrange equation can be generalized to a several variables (in our case there are *n* variables). Since there are no derivatives of *p*, the stationary functional satisfies $\partial _p L = 0$, which yields the desired result. $\square$

### Proof of Proposition 4

The pdf of *X* is

$$\begin{aligned} f_{\omega }(x) = \frac{1}{C_{\alpha }} \exp \left( -\alpha ^i \frac{d^i(x)}{i} \right) , x \in \mathbb {R}^n \end{aligned}$$

where $\alpha = \sigma ^{-1}$ is the precision. Using the variable transformation $y = \alpha x$ with Jacobian $1/\alpha ^n$ and remembering that $\alpha d(x) = d(y)$,

$$\begin{aligned} C_{\alpha } = \int _{\mathbb {R}^n} \exp \left( -\alpha ^i \frac{d^i(x)}{i} \right) \, dx = \int _{\mathbb {R}^n} \exp \left( - \frac{d^i(x)}{i} \right) \, \frac{dy}{\alpha ^n}= \frac{C_1}{\alpha ^n}. \end{aligned}$$

The moment generating function of $d^i(X)$ is

$$\begin{aligned} M_{d^i(X)}&= \int _{\mathbb {R}^n} \exp \left( z d^i(x) \right) \frac{\exp \left( - \alpha ^i \frac{d^i(x)}{i} \right) }{C_{\alpha }} \, dx \\&= \frac{C_{\root i \of {\alpha ^i/i - z} }}{C_\alpha } = \frac{1}{\left( \root i \of {1 - i\sigma ^i z} \right) ^n}, \end{aligned}$$

which coincides with the MGF of $\Gamma (n/i,i\sigma ^i)$. $\square$

### Proof of Proposition 3

From the proof of Proposition 4, it was established that the normalizing constant is $C_{\sigma _Y} = C_{1} \sigma _Y^{e-1}$ for the tropical projective torus, whose dimension is $n=e-1$.

The volume of a unit tropical sphere in the tropical projective torus $\mathbb {R}^e/\mathbb {R}{} \textbf{1}$ is equal to *e*. If the tropical radius is *r*, then the volume is $e r^{e-1}$ and hence the surface area is $e (e-1) r^{e-2}$. Therefore,

$$\begin{aligned} C_1&= \int _{\mathbb {R}^e/\mathbb {R}{} \textbf{1}} \exp {(-d_{\textrm{tr}}(x,\textbf{0}))} dx \\ {}&= \int _0^{\infty } e (e - 1) r^{e-2} \exp {(-r)} \, dr \\ {}&= e(e-1) \Gamma (e-1) = e! \end{aligned}$$

It follows that the normalizing constant is $C_{\sigma _Y} = e! \sigma _Y^{e-1}$. $\square$

### Proof of Corollary 1

Suppose that *X* comes from the Laplace or the Normal distribution, whose pdf is proportional to $\exp {(-d^i(x,\omega ^*)/(i\sigma ^{i})) }$ for $i=1$ and 2 respectively, for all $x \in \mathbb {R}^{n}$ where *d* is the Euclidean metric. Then, $X-\omega ^*$ has a distribution proportional to $\exp {(-d^i(x,\textbf{0})/(i\sigma ^{i}))}$. Clearly, $\alpha d(x,\textbf{0}) = d(\alpha x,\textbf{0})$ and so from Proposition 4, it follows that $d^i(X-\omega ^*,\textbf{0}) = d^i(X,\omega ^*) \sim i\sigma ^i \textrm{Gamma}(n/i)$. Note that for the normal distribution ($i=2$), $d^i(X,\omega ^*) \sim \sigma ^2 \chi _{n/2}$. The same argument applies for tropical Laplace and tropical Normal distributions, where the metric is tropical ($d=d_{\textrm{tr}}$), the distribution is defined on $\mathbb {R}^e/\mathbb {R}{} \textbf{1}\cong \mathbb {R}^{e-1}$ and the dimension is hence $n=e-1$. $\square$

**Prerequisites for proof of Theorem** 2

### Theorem 3

(Theorem 4.2.1 in Bierens (1996)) Let $(Q_n(\theta ))$ be a sequence of random functions on a compact set $\Theta \subset \mathbb {R}^m$ such that for a continuous real function $Q(\theta )$ on $\Theta$,

$$\begin{aligned} \sup _{\theta \in \Theta } |Q_n(\theta ) - Q(\theta )| \overset{p}{\rightarrow } 0 \text { as } n \rightarrow \infty . \end{aligned}$$

Let $\theta _n$ be any random vector in $\Theta$ satisfying $Q_n(\theta _n) = \inf _{\theta in \Theta } Q_n(\theta )$ and let $\theta _0$ be a unique point in $\Theta$ such that $Q(\theta _0) = \inf _{\theta \in \Theta } Q(\theta )$. Then $\theta _n \overset{p}{\rightarrow } \theta _0$.

### Theorem 4

(Lemma 2.4 in Newey and McFadden (1994)) If the data $z_1,\dots ,z_n$ are independent and identically distributed, the parameter space $\Theta$ is compact, $f(z_i,\theta )$ is continuous at each $\theta \in \Theta$ almost surely and there is $r(z) \ge |f(z,\theta )|$ for all $\theta \in \Theta$ and $\mathbb {E}(r(z))<\infty$, then $\mathbb {E}(f(z,\theta ))$ is continuous and

$$\begin{aligned} \sup _{\theta \in \Theta } \left| n^{-1} \sum _{i=1}^{n} f(z_i,\theta ) - \mathbb {E}(f(z,\theta )) \right| \overset{p}{\rightarrow }\ 0. \end{aligned}$$

### Lemma 1

Consider two points $x,y \in \mathbb {R}^e/\mathbb {R}{} \textbf{1}$. There exists $\eta > 0$ such that

16 $$\begin{aligned} d_{\textrm{tr}}(x+\epsilon E_i,y)= & {} d_{\textrm{tr}}(x,y) + \epsilon \phi _i(x-y),\, \forall \epsilon \in [0,\eta ], \, \forall i \in [e], \text { where }\nonumber \\ \phi _i(v)= & {} {\left\{ \begin{array}{ll} 1, &{} \text {if } v_i \ge v_j \, \forall j \in [e] \\ -1, &{} v_i < v_j \, \forall j \in [e] \backslash \{ i \} \\ 0, &{} \text {otherwise } \end{array}\right. }, \end{aligned}$$

and $E_i \in \mathbb {R}^e/\mathbb {R}{} \textbf{1}$ is a vector with 1 in the *i*-th coordinate and 0 elsewhere.

### Proof

By setting $v:=x-y$, $M:= \max _{j \in [e]} \{ v_j \}$ and $m:= \min _{j \in [e]} \{ v_j \}$,

$$\begin{aligned} d_{\textrm{tr}}(x,y)&= M - m \\ d_{\textrm{tr}}(x+\epsilon E_i,y)&= \max _{j\in [e]} \{ v_j + \epsilon \delta _{ij} \} - \min _{j\in [e]} \{ v_j + \epsilon \delta _{ij} \}, \end{aligned}$$

where $\epsilon \ge 0$, and $\delta _{ij}=\mathbb {I}(i=j)$ with $\mathbb {I}$ being the indicator function. Three separate cases are considered.

i. If $$v_i = M$$, then
  17 $$\begin{aligned} \max _{j\in [e]} \{ v_j + \epsilon \delta _{ij} \}&= v_i + \epsilon = M +\epsilon , \end{aligned}$$
  18 $$\begin{aligned} \min _{j\in [e]} \{ v_j + \epsilon \delta _{ij} \}&= m, \end{aligned}$$
  and so $d_{\textrm{tr}}(x+\epsilon E_i,y) = d_{\textrm{tr}}(x,y) + \epsilon$. Note that Eqs. (17) and (18) hold for all $\epsilon > 0$.
ii. If $v_i = m$ **and** $v_i < v_k$ for all $k \ne i$, i.e. if $v_i$ is the **unique** minimum component of vector *v*, then
  19 $$\begin{aligned} \max _{j\in [e]} \{ v_j + \epsilon \delta _{ij} \} = M,&\text { for all } \epsilon \le M-m \end{aligned}$$
  20 $$\begin{aligned} \min _{j\in [e]} \{ v_j + \epsilon \delta _{ij} \} = v_i + \epsilon = m + \epsilon ,&\text { for all } \epsilon \le m' - m, \end{aligned}$$
  where $m' {:}{=}\min _{j: v_j> m} \{ v_j \} > m$ is well-defined unless $v_j = m$ for all $j \in [e]$ i.e. for $v = m \cdot (1,\dots ,1) = \textbf{0}$, which falls under the first case. Clearly, $M \ge m'$, so for all $\epsilon \in [0, m'-m]$ Eqs. (19) and (20) are satisfied and thence $d_{\textrm{tr}}(x+\epsilon E_i,y)=d_{\textrm{tr}}(x,y) - \epsilon$.
iii. Otherwise, if none of the first two cases hold then $\exists k \ne i$ such that $m = v_k \le v_i < M$ and so
  21 $$\begin{aligned} \min _{j \in [e]} \{ v_j + \epsilon \delta _{ij} \} = v_k = m&, \text { for all } \epsilon > 0 \end{aligned}$$
  22 $$\begin{aligned} \max _{j \in [e]} \{ v_j + \epsilon \delta _{ij} \} = M&, \text { if } \epsilon \le M - v_i \end{aligned}$$
  Define $M' {:}{=}\max _{j: v_j< M} \{ v_j \}<M$ which is well-defined for all $v \ne \textbf{0}$ (first case). Since $v_i < M$, it follows by definition that $v_i \le M'$ and so $M - v_i \ge M - M' > 0$. As a result, for all $\epsilon \in [0, M - M']$, Eqs. (21) and (22) are satisfied and thence $d_{\textrm{tr}}(x+\epsilon E_i,y) = d_{\textrm{tr}}(x,y)$.

If $v=\textbf{0}$, set $\eta = + \infty$. Otherwise, for $v \ne \textbf{0}$, with $m',M'$ being well-defined, set

$$\begin{aligned} \eta = \min ( m'- m, M - M' ) > 0. \end{aligned}$$

In all three cases and for all $\epsilon \in [0,\eta ]$ the desired result is satisfied. $\square$

### Lemma 2

Consider the function $q: \mathbb {R}^e/\mathbb {R}{} \textbf{1} \rightarrow \mathbb {R}$,

$$\begin{aligned} q(x)&= \lambda _\alpha d_{\textrm{tr}}(x,\alpha ) - \lambda _\beta d_{\textrm{tr}}(x,\beta ) -\lambda _\gamma d_{\textrm{tr}}(x,\gamma ) + \lambda _\delta d_{\textrm{tr}}(x,\delta ) \\&\quad + \log \left( \frac{\lambda _\beta }{\lambda _\alpha } \right) - \log \left( \frac{\lambda _\delta }{\lambda _\gamma } \right) , \end{aligned}$$

where $\alpha ,\beta , \gamma ,\delta \in \mathbb {R}^e/\mathbb {R}\textbf{1}$, $\lambda _\alpha ,\lambda _\beta , \lambda _\gamma ,\lambda _\delta > 0$ and $(\alpha ,\lambda _\alpha ) \ne (\beta ,\lambda _\beta )$. A set $\mathcal {X}$ contains neighbourhoods of $\alpha ,\beta ,\gamma ,\delta$. If $q(x)=0,\, \forall x\in \mathcal {X}$ then $(\alpha ,\lambda _\alpha ) = (\gamma ,\lambda _\gamma )$ and $(\beta ,\lambda _\beta ) = (\delta ,\lambda _\delta )$.

### Proof

According Lemma 1, there exists $\eta _1 > 0$ such that for all $\epsilon \in [0, \eta _1]$

23 $$\begin{aligned} d_{\textrm{tr}}(x+\epsilon E_i,y) = d_{\textrm{tr}}(x,y)+ \epsilon \phi _i(x-y). \end{aligned}$$

Moreover, $d_{\textrm{tr}}(x - \epsilon E_i, y) = d_{\textrm{tr}}(y, x - \epsilon E_i) = d_{\textrm{tr}}(y+\epsilon E_i, x)$ and so using Lemma 1 again (but with *x* and *y* swapped), there exists $\eta _2>0$ such that for all $\epsilon \in [0,\eta _2]$

24 $$\begin{aligned} d_{\textrm{tr}}(x - \epsilon E_i, y) = d_{\textrm{tr}}(x,y) + \epsilon \phi _i(y-x), \end{aligned}$$

for all $\epsilon \in [0,\epsilon _0(y-x)]$. For all $\epsilon \in [0,\eta ]$ where $\eta {:}{=}\min (\eta _1,\eta _2)$, Eqs. (23), (24) are satisfied and so

$$\begin{aligned}&q(x+\epsilon E_i) = q(x) \\&\quad + \epsilon \left( \lambda _\alpha \phi _i(x-\alpha ) - \lambda _\beta \phi _i(x-\beta ) - \lambda _\gamma \phi _i(x-\gamma ) + \lambda _\delta \phi _i(x-\delta )\right) , \\&q(x-\epsilon E_i) = q(x) \\&\quad + \epsilon \left( \lambda _\alpha \phi _i(\alpha -x) - \lambda _\beta \phi _i(\beta -x) - \lambda _\gamma \phi _i(\gamma -x) + \lambda _\delta \phi _i(\delta -x) \right) . \end{aligned}$$

Consequently, for all $\epsilon \in [0,\eta ]$,

25 $$\begin{aligned}&q(x+\epsilon E_i) + q(x-\epsilon E_i) - q(x)= 0 \nonumber \\&\quad =\epsilon \left( \lambda _\alpha s_i(x-\alpha ) - \lambda _\beta s_i(x-\beta ) - \lambda _\gamma s_i(x-\gamma ) + \lambda _\delta s_i(x-\delta ) \right) , \end{aligned}$$

where

26 $$\begin{aligned} s_i(v)&{:}{=}\, \phi _i(v) + \phi _i(-v) \nonumber \\&={\left\{ \begin{array}{ll} 2, &{} \text { if } v= \textbf{0} \\ 1, &{} \text { if } v \ne \textbf{0} \text { and } v_i \text { is the non-unique maximizer or minimizer of } v \\ 0, &{} \text { otherwise } \end{array}\right. } \end{aligned}$$

By summing Eq. (25) over $i \in [e]$ and defining $s(v) = \sum _{i=1}^e s_i(v)$,

27 $$\begin{aligned} \lambda _\alpha s(x-\alpha ) - \lambda _\beta s(x-\beta ) - \lambda _\gamma s(x-\gamma ) + \lambda _\delta s(x-\delta ) = 0, \end{aligned}$$

$$\forall x \in \mathcal {X}$$.

Here we try to prove by contradiction that $\mathcal {S}:= \{\alpha ,\delta \}\cap \{\gamma ,\beta \}$ is not empty. Suppose that $\mathcal {S}:= \{\alpha ,\delta \}\cap \{\gamma ,\beta \} = \emptyset$. Then, setting $x= \alpha$ in Eq. (27) and noting that $s(0) = 2e$ and $0 \le s(v) \le e$ for $v \ne 0$, we get $2e \lambda _\alpha \le e \lambda _\beta + e \lambda _\gamma$, since $\beta , \gamma \ne \alpha$. Applying the same argument to $x=\beta ,\gamma ,\delta$, the following system of inequalities holds

$$\begin{aligned} 2\lambda _\alpha&\le \lambda _\beta + \lambda _\gamma \\ 2\lambda _\beta&\le \lambda _\alpha + \lambda _\delta \\ 2\lambda _\gamma&\le \lambda _\alpha + \lambda _\delta \\ 2\lambda _\delta&\le \lambda _\beta + \lambda _\gamma . \end{aligned}$$

It follows that $\lambda _\alpha = \lambda _\beta = \lambda _\gamma =\lambda _\delta$. Then, rewrite Eq. (27) as

28 $$\begin{aligned} s(x-\alpha ) - s(x-\beta ) - s(x-\gamma ) + s(x-\delta ) = 0, \end{aligned}$$

Note now Eq. (28) can only hold at $x=\alpha$ iff $s(\alpha - \gamma ) = s(\alpha -\beta ) = e$ and $s(\alpha -\delta )=0$. But $s(v)=e$ if and only if all the components of *v* are non-unique minimizers and maximizers or $\{v_i:i\in [e]\}=\{\zeta ,\kappa \}$, where $\zeta < \kappa$ and $|\{i:v_i = \zeta \}|=n_\zeta , |\{i: v_i = \kappa \}| = n_\kappa$, such that $n_\zeta + n_\kappa = e$ and $n_\zeta , n_\kappa \ge 2$.

Consider $z =v+\epsilon E_i$, where $v_i = \zeta$ and $0<\epsilon < \kappa -\zeta$. The minimum and maximum components of *z* are $\zeta$ and $\kappa$, and $\{z_i:i\in [e]\}=\{\zeta ,\zeta + \epsilon , \kappa \}$ with $|\{i:z_i = \zeta \}|=n_\zeta - 1,|\{i: z_i = \kappa \}| = n_\kappa$. It follows that,

$$\begin{aligned} s(z) = |\{i:z_i = \zeta \}| +|\{i: z_i = \kappa \}| = e-1. \end{aligned}$$

Now consider $z = v + \epsilon E_i$ where $v_i = \kappa$. The maximum is no longer unique, but the $n_{\zeta }$ minima are still unique. Therefore, $s(z) = n_{\zeta } \ge 2$. Combining the two cases, it is concluded that $s(v + \epsilon E_i) \ge 2$ for all $i \in [e]$.

Set $x = \alpha + \epsilon E_i$, where $\alpha _i - \beta _i = \min _k \{ \alpha _k - \beta _k \}$. Then,

29 $$\begin{aligned} s(x - \alpha ) = s(\epsilon E_i)= e-1, \end{aligned}$$

since there is a unique maximizer, but all the other $e-1$ components are 0, which is the minimum. Furthermore,

30 $$\begin{aligned} s(x - \beta ) = s(\alpha - \beta + \epsilon E_i) = e-1, \end{aligned}$$

since for $v = \alpha - \beta$ with $s(v) = e$, it corresponds to the first case examined. It is assumed that $\epsilon < \kappa - \zeta = d_{\textrm{tr}}(\alpha - \beta )$. Moreover,

31 $$\begin{aligned} s(x - \gamma ) = s(\alpha -\gamma + \epsilon E_i) \ge 2, \end{aligned}$$

for $v = \alpha - \gamma$ with $s(v) = e$. Finally, since $s(\alpha - \delta ) = 0$ and so the components of $\alpha - \delta$ have a unique minimum and a unique maximum, there exists a neighborhood around $x = \alpha$ such that $x-\alpha$ still has that property, i.e.

32 $$\begin{aligned} s(x - \delta ) = s(\alpha - \delta + \epsilon E_i) = 0 \end{aligned}$$

for all $\epsilon < \eta$ for some $\eta >0$.

From Eqs. (29)–(32), it is concluded that

33 $$\begin{aligned} s(x-\alpha ) - s(x-\beta ) - s(x-\gamma ) + s(x-\delta ) \le -2 , \end{aligned}$$

which contradicts Eq. (28). Therefore $\mathcal {S} = \{\alpha ,\delta \}\cap \{\gamma ,\beta \} \ne \emptyset$.

Define another set $\mathcal {T} = \{\alpha ,\beta ,\gamma ,\delta \}$. Since $\mathcal {S} \ne \emptyset$, $|\mathcal {T}| \le 3$. Suppose that $|\mathcal {T}| = 3$ with $\mathcal {T} = \{\tau , \upsilon ,\phi \}$. Then, without loss of generality Eq. (27) becomes

34 $$\begin{aligned} \lambda _\tau s(x-\tau ) + \lambda _\upsilon s(x- \upsilon ) - \lambda _\phi s(x-\phi ) = 0 \end{aligned}$$

Similarly to before, setting $x=\tau ,\upsilon ,\phi$ yields,

$$\begin{aligned} 2\lambda _\tau&\le \lambda _\phi \\ 2\lambda _\upsilon&\le \lambda _\phi \\ 2\lambda _\phi&\le \lambda _\tau + \lambda _\upsilon , \end{aligned}$$

which is contradictory since $\lambda _\tau + \lambda _\upsilon >0$. Therefore, $|\mathcal {T}|\le 2$. There are 4 cases to consider

i. $$\alpha = \delta \ne \beta = \gamma $$, but then $$\mathcal {S} = \emptyset $$,
ii. $\alpha = \beta \ne \gamma = \delta$, but then Eq. (27) can only be satisfied $x=\alpha ,\gamma$ if $\lambda _\alpha = \lambda _\beta$ and $\lambda _\gamma = \lambda _\delta$ which violates the statement that $(\alpha ,\lambda _\alpha ) \ne (\beta ,\lambda _\beta )$,
iii. $\alpha = \gamma \ne \beta = \delta$ and from Eq. (27) at $x=\alpha ,\gamma$ it follows that $\lambda _\alpha = \lambda _\gamma , \lambda _\beta = \lambda _\delta$ and hence the desired result,
iv. $\alpha = \beta =\gamma =\delta$, in which case
  $$\begin{aligned} q(x) = (\lambda _\alpha - \lambda _\beta - \lambda _\gamma + \lambda _\delta ) d_{\textrm{tr}}(x,\alpha ) +\log \left( \frac{\lambda _\beta }{\lambda _\alpha } \right) - \log \left( \frac{\lambda _\delta }{\lambda _\gamma } \right) , \end{aligned}$$
  which can only be uniformly 0 at $\mathcal {X}$ if and only if $\lambda _\alpha + \lambda _\delta = \lambda _\beta + \lambda _\gamma$. Observe that $(\lambda _\alpha ,\lambda _\delta )$ and $(\lambda _\beta , \lambda _\gamma )$ are the two roots of the same quadratic $z^2 - (\lambda _\alpha + \lambda _\delta ) z + \lambda _\alpha \lambda _\delta$ and noting that in this case $\lambda _\alpha \ne \lambda _\beta$, it follows that $\lambda _\alpha = \lambda _\gamma$ and $\lambda _\beta = \lambda _\delta$.

$$\square $$

### Lemma 3

Consider a compact set $\Sigma \subseteq \mathbb {R}_+ = (0,\infty )$. Then the set $\Lambda = \{ \sigma ^{-1}: \sigma \in \Sigma \} \in \mathbb {R}_+$ is also compact.

### Proof

In metric spaces, a set is compact iff it is sequentially compact. Therefore, for every sequence $\sigma _n \in \Sigma$, $\sigma _n \rightarrow \sigma \in \Sigma$. Every sequence in $\Lambda$ can be expressed as $1/\sigma _n$, which tends to $1/\sigma \in \Lambda$. Therefore, $\Lambda$ is sequentially compact and hence compact. $\square$

### Proof of Theorem 2

This proof has been written for precision estimators $\lambda =1/\sigma$ instead of deviation estimators. For the rest of the proof consider $\lambda _y = \sigma _y^{-1}$ for $y=0,1$ and define the set

$$\begin{aligned} \Lambda = \{ \sigma ^{-1}: \sigma \in \Sigma \} \in \mathbb {R}_+. \end{aligned}$$

According to Lemma 3, $\Lambda$ is also compact.

Define the functions *f* and *h* as

$$\begin{aligned} f: \mathbb {R}^e/\mathbb {R}{} \textbf{1}&\times \{0,1\} \times \Omega ^2 \times \Lambda ^2 \rightarrow \mathbb {R}, \\ f((x,y),(\omega ,\lambda ) )&= y \log S(h(x,(\omega ,\lambda ))) +(1-y) \log S(-h(x,(\omega ,\lambda ))), \\ h: \mathbb {R}^e/\mathbb {R}{} \textbf{1}&\times \Omega ^2 \times \Lambda ^2 \rightarrow \mathbb {R},\\ h(x,(\omega ,\lambda ))&= \lambda _0 d_{\textrm{tr}}(x,\omega _0) - \lambda _1 d_{\textrm{tr}}(x,\omega _1) +(e-1) \log {\frac{\lambda _{1} }{\lambda _0}}, \end{aligned}$$

where *S* is the logistic function. Also denote the empirical ($Q_n$) and expected (*Q*) log-likelihood functions as

$$\begin{aligned} Q_n(\omega ,\lambda )&= \frac{1}{n} \sum _{i=1}^{n} f((X_i,Y_i),(\omega ,\lambda )) ~\text { with }\\ Q_n(\hat{\omega }_n,\hat{\lambda }_n)&= \sup _{\omega \in \Omega ^2, \lambda \in \Lambda ^2 } Q_n(\omega ),~\text { and }\\ Q(\omega ,\lambda )&= \mathbb {E}_{(X,Y)} \left( f((X,Y),(\omega ,\lambda )) \right) \\&= \mathbb {E}_X\bigg ( S(h(X,(\omega ^*,\lambda ^*) )) \log ( S(h(X,(\omega ,\lambda ) ) )) \\&\quad + S(-h(X,(\omega ^*,\lambda ^*) )) \log ( S(-h(X,(\omega ,\lambda ) ) ) ) \bigg ). \end{aligned}$$

The last equation follows from conditioning on

$$\begin{aligned} Y \sim \text {Bernoulli}(S(h(X,(\omega ^*,\lambda ^*) ))). \end{aligned}$$

Before we move on, we need to prove that $f((X,Y),(\omega ,\lambda ))$ is integrable so that *Q* is well-defined. Without loss of generality assume that $\lambda _1 \ge \lambda _0$. It suffices to prove that $\mathbb {E}(f((X,Y),(\omega ,\lambda )),Y=y)$ is integrable for both $y=0,1$. Observe that

$$\begin{aligned} h(X,(\omega ,\lambda ))&\le (\lambda _0 -\lambda _1) d_{\textrm{tr}}(X,\omega _0) + \lambda _1 d_{\textrm{tr}}(\omega _0,\omega _1) + \text {const} \\&\le \lambda _1 d_{\textrm{tr}}(\omega _0,\omega _1) + \text {const}. \end{aligned}$$

Since $h(X,(\omega ,\lambda ))$ is bounded above, $f((X,Y),(\omega ,\lambda ))$ is also bounded below on $Y=0$ and is hence integral on $Y=0$. Also, observe that

$$\begin{aligned} h(X,(\omega ,\lambda )) \ge (\lambda _0 -\lambda _1) d_{\textrm{tr}}(X,\omega _1) - \lambda _0 d_{\textrm{tr}}(\omega _0,\omega _1) + \text {const} \end{aligned}$$

and noting that $\log (S(x)) > x-1$ for all $x<0$

$$\begin{aligned} \log (S(h(X,(\omega ,\lambda )))) \ge h(X,(\omega ,\lambda )) - 1 \ge (\lambda _0 -\lambda _1) d_{\textrm{tr}}(X,\omega _1) +\text {const}. \end{aligned}$$

Since $d_{\textrm{tr}}(X,\omega _1)$ is integrable on $Y=1$, the LHS is integrable on $Y=1$ too. It follows that $f(X,(\omega ,\lambda ))$ is integrable and hence *Q* is well-defined.

First, we prove that *Q* is maximised at $(\omega ,\lambda ) = (\omega ^*,\lambda ^*)$ and that this maximizer is unique. Consider the function

$$\begin{aligned} g: \mathbb {R} \rightarrow \mathbb {R}, \, g(t) = S(\alpha ) \log S(t) + S(-\alpha ) \log S(-t), \end{aligned}$$

where $\alpha \in \mathbb {R}$ is some constant. The function *g* is maximised at $t=\alpha$ and applying Taylor’s theorem yields

$$\begin{aligned} g(x) = g(\alpha ) - \frac{1}{2} S(\xi ) S(-\xi ) (x-\alpha )^2, \text { for some } \xi \in (\alpha ,x). \end{aligned}$$

Setting $\alpha = h(X,(\omega ^*,\lambda ^*) )$ and denoting $\xi$ as a random variable

$$\begin{aligned} \xi (X) \in (h(X,(\omega ^*,\lambda ^*)),h(X,(\omega ,\lambda ))) \end{aligned}$$

observe that

35 $$\begin{aligned} Q(\omega ,\lambda )&= \mathbb {E}_X( g(h(X,(\omega ,\lambda ) ) ) ) \nonumber \\&= \mathbb {E}_X ( g(h(X,(\omega ^*,\lambda ^*) ) ) - \frac{1}{2} \mathbb {E}_X \left( S(\xi (X)) S(-\xi (X)) [h(X,(\omega ,\lambda ) )- h(X,(\omega ^*,\lambda ^*) )]^2 \right) \nonumber \\&\le Q(\omega ^*,\lambda ^*), \end{aligned}$$

Hence, from the expression above it is deduced that $(\omega ^*,\lambda ^*)$ is a maximizer. Now, consider the function $q: \mathcal {X} \rightarrow \mathbb {R}$

$$\begin{aligned} q(x) = h(x,(\omega ^*,\lambda ^*) ) - h(x,(\omega ,\lambda )), \end{aligned}$$

where $\Omega \subset \mathcal {X} \subset \mathbb {R}^e/\mathbb {R}{} \textbf{1}$ such that for some $\zeta >0$

$$\begin{aligned} \mathcal {X} = \{x \in \mathbb {R}^e/\mathbb {R}{} \textbf{1}: \inf _{\omega \in \Omega } d_{\textrm{tr}}(x,\omega )<\zeta \}, \end{aligned}$$

so that for any $\omega \in \Omega$ there is a neighborhood of $\omega$ within $\mathcal {X}$. Note that $\mathcal {X}$ is a bounded set since $\Omega$ is bounded too.

We will prove by contradiction that $q(x) = 0, \forall x \in \mathcal {X}$. Suppose there exists $x_0 \in \mathcal {X}$ such that $q(x_0) > 0$, then since *q* is continuous there exists a neighborhood *U* with $x_0 \in U$ such that $q(x) > 0$ for all $x \in U$ and so

$$\begin{aligned} \mathbb {E}(q^2(X) \mathbb {I}(X \in U) ) >0, \end{aligned}$$

where $\mathbb {I}$ is the indicator function. Since $h(x,(\omega ,\lambda ))$ is continuous with respect to *x* and $\mathcal {X}$ is bounded, the function takes values on a bounded interval and hence $\xi (x)$ is bounded in $\mathcal {X}$ i.e. there exists $\epsilon >0$ such that $\mathbb {P}(S(\xi (X) S(-\xi (X)) > \epsilon | X \in U) = 1$ and so Eq. (35) becomes

$$\begin{aligned} Q(\omega ,\lambda ) \le Q(\omega ^*,\lambda ^*) - \frac{\epsilon }{2} \mathbb {E}(q^2(X) \mathbb {I}(X \in U) ) < Q(\omega ^*,\lambda ^*), \end{aligned}$$

since $\mathbb {P}(X \in U) >0$ (*X* has positive density everywhere). Therefore, for $(\omega ,\lambda )$ to be a maximizer, $q(x) = 0$ for all $x \in \mathcal {X}$. Apply Lemma 2 with $\omega ^* = (\alpha ,\beta )$, $\omega = (\gamma ,\delta )$, $\lambda ^* = (\lambda _\alpha ,\lambda _\beta )$ and $\lambda = (\lambda _\gamma ,\lambda _\delta )$ with the set $\mathcal {X}$ containing neighbourhoods of $\alpha ,\beta ,\gamma ,\delta$ and $q(x) = 0$ for all *x* in those neighbourhoods. It is concluded that $\omega = \omega ^*$ and $\lambda = \lambda ^*$, thus proving the uniqueness of the maximizer.

Theorem 4 provides the uniform law of large numbers. The parameter space $\Omega ^2 \times \Lambda ^2$ is compact since $\Omega$ and $\Lambda$ are compact. Moreover, $f((x,y),(\omega ,\lambda ) )$ is clearly continuous at each $(\omega ,\lambda ) \in \Omega ^2 \in \Lambda ^2$. Finally, consider the function

$$\begin{aligned} r(z) = \sup _{\omega \in \Omega ^2,\lambda \in \Lambda ^2} \{ |f(z,(\omega ,\lambda ) )| \} = - f(z, \omega (z), \lambda (z)), \end{aligned}$$

since *f* is non-positive. The function $\omega (z), \lambda (z)$ are chosen to minimize *f*. Using Eq. (35),

$$\begin{aligned} \mathbb {E}(r(X))&\le - Q(\omega ^*,\lambda ^*) + \frac{1}{2} \mathbb {E}( \left[ h(X,(\omega (X),\lambda (X)))- h(X,(\omega ^*,\lambda ^*)) \right] ^2) , \end{aligned}$$

since the sigmoid function is bounded by 1. Note that

$$\begin{aligned} \mathbb {E}((Z+W)^2) \le 2(\mathbb {E}(Z^2) +\mathbb {E}(W^2)), \end{aligned}$$

and set $W = \log (\lambda _1(X)/\lambda _0(X)) - \log (\lambda _1^*/\lambda _0^*)$. Since $\lambda _y(X) \in \Lambda \subseteq [a,b]$ for some $b\ge a>0$, it follows that $W^2$ is integrable and so now we just have to prove that *Z* is integrable, where $Z=Z_1+Z_2+Z_3+Z_4$ with the four terms corresponding to tropical distance function $\lambda d_{\textrm{tr}}(X,\omega )$. It also holds

$$\begin{aligned} \mathbb {E}((Z_1+Z_2+Z_3+Z_4)^2) \le 2(\mathbb {E}(Z_1^2) +\mathbb {E}(Z_2^2) + \mathbb {E}(Z_3^2) +\mathbb {E}(Z_4^2)) \end{aligned}$$

and so $\mathbb {E}(Z^2)$ is bounded above by

$$\begin{aligned}&\mathbb {E}\left( \sum _{i=0}^1 \lambda _i^2 d_{\textrm{tr}}^2(X,\omega _i(X))) + (\lambda _i^*)^2 d_{\textrm{tr}}^2(X,\omega ^*_i(X))\right) \\&\quad \le \mathbb {E}_Y \left[ 2 \left( \sum _{i=0}^1 \lambda _i^2 + (\lambda _i^*)^2 \right) \mathbb {E}\left( d_{\textrm{tr}}^2(X,\omega ^*_Y)|Y \right) \right. \left. + 2 \left( \sum _{i=0}^1 \lambda _i^2 d_{\textrm{tr}}^2( \omega _i(X),\omega _Y^*) + (\lambda _i^*)^2 d_{\textrm{tr}}^2(\omega ^*_i,\omega _Y^*) \right) \right] , \end{aligned}$$

where the second inequality came from applying the triangular inequality four times in the form $d_{\textrm{tr}}(X,\tau ) \le d_{\textrm{tr}}(X,\omega ^*_Y) + d_{\textrm{tr}}(\omega ^*_Y,\tau )$. The final expression is finite because $\Omega$ is compact and hence $d_{\textrm{tr}}( \omega _i(X),\omega _Y^*)$ is finite, $d_{\textrm{tr}}(X,\omega _Y)|Y$ is square-integrable. Therefore, $\mathbb {E}(r(X))$ is finite.

All conditions of the theorem are satisfied and so

$$\begin{aligned} \sup _{\omega \in \Omega ^2} \left| \frac{1}{n} \sum _{i=1}^n f( (X_i,Y_i), \omega ) - \mathbb {E}(f( (X,Y), \omega )) \right| = \sup _{\omega \in \Omega ^2} \left| Q_n(\omega ) - Q(\omega ) \right| \overset{p}{\rightarrow }\ 0. \end{aligned}$$

Finally, using Theorem 3 and combining the uniqueness of the maximizer with the uniform bound result, it is concluded that $\hat{\omega } \overset{p}{\rightarrow } \omega ^*$. $\square$

### Proof of Proposition 5

First, define $\Delta _0 = \{ C(X) \ne 1 | Y = 0 \}$. By definition of *C*(*X*),

$$\begin{aligned} \Delta _0&= \left\{ \left. (\sigma _0^{-1} - \sigma _1^{-1}) d_\textrm{tr}(X,\hat{\omega }) - (e-1) \log {\left( \frac{\sigma _1}{\sigma _0} \right) } \ge 0 \right| Y=0 \right\} \\&= \left\{ \left. d_\textrm{tr}(X,\hat{\omega }) \ge \alpha \sigma _0 \sigma _1 \right| Y=0 \right\} . \end{aligned}$$

Triangular inequality dictates that

$$\begin{aligned} d_\textrm{tr}(X,\omega ^*) - d_\textrm{tr}(\omega ^*,\hat{\omega }) \le d_\textrm{tr}(X,\hat{\omega }) \le d_\textrm{tr}(X,\omega ^*) + d_\textrm{tr}(\omega ^*,\hat{\omega }), \end{aligned}$$

and so it follows that

$$\begin{aligned} \Delta _0&\supseteq \{ d_\textrm{tr}(X,\omega ^*) \ge \sigma _0 \sigma _1 \left( \alpha + \epsilon \right) | Y = 0 \} \\ \Delta _0&\subseteq \{ d_\textrm{tr}(X,\omega ^*) \ge \sigma _0 \sigma _1 \left( \alpha - \epsilon \right) | Y = 0 \}, \end{aligned}$$

and since $$Z = \sigma _0^{-1} d_\textrm{tr}(X,\omega ^*)|Y=0\sim F$$,

$$\begin{aligned} \mathbb {P}( Z \ge \sigma _1 (\alpha + \epsilon )) \le \mathbb {P}(\Delta _0) \le \mathbb {P}( Z \ge \sigma _1 (\alpha - \epsilon )), \end{aligned}$$

which yields the desired result.

Similarly, for $$\Delta _1 = \{ C(X) \ne 0 | Y = 1 \} = \left\{ d_\textrm{tr}(X,\hat{\omega }) \le \sigma _0 \sigma _1 \alpha \right\} $$,

$$\begin{aligned} \Delta _1&\supseteq \{ d_\textrm{tr}(X,\omega ^*) \le \sigma _0 \sigma _1 \left( \alpha - \epsilon \right) | Y = 1 \} \\ \Delta _1&\subseteq \{ d_\textrm{tr}(X,\omega ^*) \le \sigma _0 \sigma _1 \left( \alpha + \epsilon \right) | Y = 1 \}, \end{aligned}$$

and since $$Z = \sigma _1^{-1} d_\textrm{tr}(X,\omega ^*)|Y=1\sim F$$,

$$\begin{aligned} \mathbb {P}( Z \le \sigma _0 (\alpha - \epsilon )) \le \mathbb {P}(\Delta _1) \le \mathbb {P}( Z \le \sigma _0 (\alpha + \epsilon )), \end{aligned}$$

which is the desired interval.

For the second part of the proposition, $\hat{\omega } = \omega ^*$ and so $\epsilon = 0$. Hence,

$$\begin{aligned} \mathbb {P}(\Delta _0)&= 1 - F(\sigma _1 \alpha ) = 1- F( x u(x) ) \\ \mathbb {P}(\Delta _1)&= F(\sigma _0 \alpha ) = F( u(x) ), \text { where } \\ x = \frac{\sigma _1}{\sigma _0}&\text { and } u(x) = (e-1) \frac{ \log {x} }{x - 1} \end{aligned}$$

Consider the function

$$\begin{aligned} g(x) = 1-F(xu(x)) - F(u(x)) \end{aligned}$$

Proving that $$g(x) <0 $$ for all $$x > 1$$ is equivalent to proving the desired result that $$\mathbb {P}(\Delta _0) < \mathbb {P}(\Delta _1)$$ for $$\sigma _1 > \sigma _0$$. First,

$$\begin{aligned} \lim _{x \rightarrow 1} u(x) = \lim _{x \rightarrow 1} x u(x) = e-1, \end{aligned}$$

and so $\lim _{x \rightarrow 1} g(x) = 1-2F(e-1)$. It is a well-known fact that the median of the $\textrm{Gamma}$ distribution is less than the mean. Hence, for $Z \sim \textrm{Gamma}(e-1,1)$ with mean $e-1$, $F(e-1) > \frac{1}{2}$ and so

36 $$\begin{aligned} \lim _{x \rightarrow 1} g(x) < 0. \end{aligned}$$

Finally, the derivative of *g* is

$$\begin{aligned} g'(x) = - F'(u(x)) u'(x) - F'(xu(x)) (xu'(x) + u(x)) \end{aligned}$$

The following two inequalities

37 $$\begin{aligned} F'(u(x))&\ge F'(xu(x)), \end{aligned}$$

38 $$\begin{aligned} u'(x) + xu'(x) + u(x)&\ge 0, \end{aligned}$$

imply that

39 $$\begin{aligned} g'(x) \le - F'(xu(x)) (u'(x) + xu'(x) + u(x)) \le 0. \end{aligned}$$

From (36) and (39) it follows that $g(x) < 0$ for all $x>1$.

For inequality (37), remember that

$$\begin{aligned} F'(x) = \frac{x^{e-2} \exp {(-x)} }{\Gamma (e-1)} \end{aligned}$$

and so

$$\begin{aligned} F'(u(x)) - F'(xu(x))&= F'(u(x)) \left( 1 - x^{e-2} \exp (-(x-1) u(x)) \right) \\ {}&= F'(u(x)) \left( 1 - x^{e-2} \exp (-(e-1) \log (x)) \right) \\ {}&= F'(u(x))(1-x^{-1})>0, \end{aligned}$$

for all $$x>1$$.

For inequality (38),

$$\begin{aligned} u'(x) + xu'(x) + u(x) = \frac{e-1}{(x-1)^2} \left( x-x^{-1} -2\log {x} \right) , \end{aligned}$$

is a non-negative function for $x>1$ iff *v* is a non-negative function, where

$$\begin{aligned} v(x)&= x - x^{-1} - 2\log {x}, \text { with } \\ v'(x)&= \frac{(x-1)^2}{x^2} \ge 0 \text { and } v(1) = 0. \end{aligned}$$

Clearly, *v* is a non-negative function for $x>1$, so inequality (38) is satisfied. $\square$

### Proof of Proposition 6

For symbolic convenience, in this proof class 0 is referred to as class $-1$ and so $Y \in \{-1,1\}$. Applying the triangular inequality twice,

$$\begin{aligned} D_X&= d_\textrm{tr}(X , \omega ^*_Y) - d_\textrm{tr}(X , \omega ^*_{-Y})\\&\ge \left( d_\textrm{tr}(X , \hat{\omega }_Y) - d_\textrm{tr}( \omega ^*_Y , \hat{\omega }_Y ) \right) \\&\quad - \left( d_\textrm{tr}(X , \hat{\omega }_{-Y}) + d_\textrm{tr}( \omega ^*_{-Y} , \hat{\omega }_{-Y})\right) \\&= d_\textrm{tr}(X , \hat{\omega }_Y) - d_\textrm{tr}(X , \hat{\omega }_{-Y}) - \epsilon , \end{aligned}$$

it follows that

$$\begin{aligned} \{ C(X) \ne Y \} = \{d_\textrm{tr}(X, \hat{\omega }_Y) - d_\textrm{tr}(X, \hat{\omega }_{-Y}) \ge 0\} \subseteq \{D_X \ge - \epsilon \} \end{aligned}$$

and so the generalization error has the following upper bound

40 $$\begin{aligned} \mathbb {P}( C(X) \ne Y ) \le \mathbb {P} \left( D_X \ge -\epsilon \right) . \end{aligned}$$

Note that if $d_\textrm{tr}(X,\omega _Y^*) < \Delta _\epsilon$, then by the use of triangular inequality

$$\begin{aligned} D_X&=d_\textrm{tr}(X , \omega ^*_Y) - d_\textrm{tr} (\omega ^*_{-Y},X)\\&\le d_\textrm{tr}(X , \omega ^*_Y) - \left( d_\textrm{tr}(\omega _{-Y}^*, \omega _Y^* ) -d_\textrm{tr} (\omega ^*_Y,X) \right) \\&< 2\Delta _{\epsilon }-d_\textrm{tr}(\omega _1^* , \omega _{-1}^*) = -\epsilon . \end{aligned}$$

Consequently,

41 $$\begin{aligned} \mathbb {P}( C(X) \ne Y ) \le \mathbb {P}\left( D_X \ge -\epsilon , Z_X \ge \Delta _\epsilon \right) \end{aligned}$$

Since the distribution of *X* is symmetric around $\omega ^*_Y$, the random variable $2\omega ^*_Y - X$ has the same distribution and so

42 $$\begin{aligned} \mathbb {P}\left( D_X \ge -\epsilon , Z_X \ge \Delta _\epsilon \right) = \mathbb {P}\left( D_{2\omega _Y^*-X} \ge -\epsilon , Z_{2\omega _Y^*-X} \ge \Delta _\epsilon \right) . \end{aligned}$$

It will be proved that

43 $$\begin{aligned} Z_{2\omega _Y^*-X}&= Z_{X}, \end{aligned}$$

44 $$\begin{aligned} D_X + D_{2\omega _Y^*-X}&\le 0, \end{aligned}$$

and so $\{D_{2\omega _Y^*-X} \ge -\epsilon , Z_{2\omega _Y^*-X} \ge \Delta _\epsilon \} \subseteq \{D_{X} \le \epsilon , Z_{X} \ge \Delta _\epsilon \}.$ Then, using Eq. (42),

$$\begin{aligned} \mathbb {P}\left( D_X \ge -\epsilon , Z_X \ge \Delta _\epsilon \right) \le \mathbb {P}\left( D_{X} \le \epsilon , Z_{X} \ge \Delta _\epsilon \right) , \end{aligned}$$

and substituting it to inequality (41),

$$\begin{aligned} \mathbb {P}(C(X) \ne Y)) =&\frac{1}{2} ( \mathbb {P}\left( D_X \ge -\epsilon , Z_X \ge \Delta _\epsilon \right) \\&+\mathbb {P}\left( D_X \le \epsilon , Z_X \ge \Delta _\epsilon \right) ) \\ =&\mathbb {P}\left( Z_X \ge \Delta _\epsilon \right) + h(\epsilon ) \end{aligned}$$

where $h(\epsilon ) = \mathbb {P}(Z_X \ge \Delta _\epsilon , |D_X| \le \epsilon )$ is an increasing function with respect to $\epsilon$, which completes the first part of the proof.

Equation (43) follows from the observation that

$$\begin{aligned} d_\textrm{tr}(2\omega _Y^*-x,\omega _Y^*) = d_\textrm{tr}(x,\omega _Y^*). \end{aligned}$$

For Eq. (44),

$$\begin{aligned} D_{2\omega _Y^*-X} + D_X&= Z_{2\omega _Y^*-X} - d_\textrm{tr}( 2 \omega _Y^* - X , \omega _{-Y}^* ) \\&\quad + Z_X - d_\textrm{tr}(X,\omega _{-Y}^*) \\&\overset{\textrm{A28}}{=} 2Z_{2\omega _Y^*-X} - d_\textrm{tr}( 2 \omega _Y^* - X, \omega _{-Y}^* ) - d_\textrm{tr}(\omega _{-Y}^*,X)\\&\le 2Z_{2\omega _Y^*-X} - d_\textrm{tr}( 2 \omega _Y^* - X, X ) = 0, \end{aligned}$$

where the last inequality comes from the triangular inequality. Finally, the consistency of the learning algorithm is proved. Under the conditions of Theoreom 2, the maximum likelihood estimator $\hat{\omega } = (\hat{\omega }_0, \hat{\omega }_1) \overset{\textrm{p}}{\rightarrow }(\omega _0^*,\omega _1^*)$ as $n \rightarrow \infty$ where $(X_1,Y_1), \dots , (X_n,Y_n)$ is the sample. For the rest of the proof, the test covariate-response pair (*X*, *Y*) is independent from the afore training sample. Define the classifier,

$$\begin{aligned} C_{\omega }(x) = \textrm{sgn}\left( d_\textrm{tr}(x,\omega _0) - d_\textrm{tr}(x,\omega _1) \right) \end{aligned}$$

where $\omega = (\omega _0,\omega _1)$. The Bayes predictor is $C_{\omega _0^*,\omega _1^*}$. Noting that $C_{\omega _0^*,\omega _1^*}(X) = \textrm{sgn}(D_X)Y$, the Bayes (or irreducible) error is

$$\begin{aligned} \textrm{BE} = \mathbb {P}( \textrm{sgn}(D_X)Y \ne Y ) = \mathbb {P}( D_X > 0) = \mathbb {P}( D_X \ge 0), \end{aligned}$$

since it is assumed that $\mathbb {P}(D_X =0)=0$. Using inequality 40 derived earlier, it follows that the generalization error is bounded by

$$\begin{aligned} \mathbb {P}( D_X \ge 0) = \textrm{BE} \le \mathbb {P}(C_{\hat{\omega }}(X) \ne Y) \le \mathbb {P}(D_X \ge -\epsilon (\hat{\omega })), \end{aligned}$$

where $\epsilon (\hat{\omega }_0,\hat{\omega }_1)= d_\textrm{tr}(\omega _0,\omega _0^*) + d_\textrm{tr}(\omega _1,\omega _1^*) \overset{\textrm{p}}{\rightarrow }\ 0$. as the training sample size $n \rightarrow \infty$ according to Theorem 2. The complementary CDF of $D_X$, defined as

$$\begin{aligned} F_{D_X}^C(x) = \mathbb {P}(D_X \ge x), \end{aligned}$$

is a continuous function and so it follows that $F_{D_X}^C(\epsilon (\hat{\omega })) \overset{\textrm{p}}{\rightarrow }\ F_{D_X}^C(0) = \textrm{BE}$ as $n \rightarrow \infty$. From the probability squeeze theorem,

$$\begin{aligned} \mathbb {P}(C_{\hat{\omega }}(X)\ne Y| (X_1,Y_1), \dots , (X_n,Y_n) ) \overset{\textrm{p}}{\rightarrow }\ \textrm{BE} \text { as } n \rightarrow \infty . \end{aligned}$$

This concludes the proof of the consistency of the algorithm. $\square$

### Proof of Proposition 7

Consider the random variable $d_\textrm{tr}(X,\alpha )$. From the triangular inequality

$$\begin{aligned} d_\textrm{tr}(X,\alpha ) \le d_\textrm{tr}(X,\omega ^*) + d_\textrm{tr}(\alpha ,\omega ^*), \end{aligned}$$

it is deduced that $d_\textrm{tr}(X,\alpha )$ is integrable, bounded above by an integrable random variable.

Now consider the function $F: \mathbb {R}^e/\mathbb {R}{} \textbf{1}\rightarrow \mathbb {R}$,

$$\begin{aligned} F(x) = d_\textrm{tr}(x,\omega ) + d_\textrm{tr}(2\omega ^* - x,\omega ) - 2d_\textrm{tr}(x,\omega ^*). \end{aligned}$$

Noting that $d_\textrm{tr}(2\omega ^* - x,\omega ) = d_\textrm{tr}(x,2\omega ^* - \omega )$, it follows that *F*(*X*) is integrable as the sum of integrable random variables.

From triangular inequality and the fact that $d_\textrm{tr}(2\omega ^* - x,x) = 2d_\textrm{tr}(x,\omega ^*)$ it follows that $F(x) \ge 0$ for all $x \in \mathbb {R}^e/\mathbb {R}{} \textbf{1}$. Furthermore, $F(\omega ^*)>0$ and since *F* is continuous, there exists a neighbourhood *U* that contains $\omega ^*$ such that $F(x) > 0$ for all $x \in U$. Moreover, the function has positive density in a neighbourhood *V* that contains the centre $\omega ^*$. Therefore, there exists a neighbourhood $W = U \cap V$ such that $F(x) > 0$ for all $x \in W$ and $\mathbb {P}(X \in W) > 0$. Hence, since $F(X) \ge 0$,

$$\begin{aligned} \mathbb {E}(F(X)) \ge \mathbb {E}(F(X)| X \in W) \mathbb {P}(X \in W ) > 0. \end{aligned}$$

In other words,

45 $$\begin{aligned} \mathbb {E}\left( d_\textrm{tr}(X,\omega )\right) + \mathbb {E}\left( d_\textrm{tr}(2\omega ^*-X,\omega )\right) > 2 \mathbb {E}(d_\textrm{tr}(x,\omega ^*)) \end{aligned}$$

Moreover, consider the isometry $y = 2\omega ^*-x$ and note that for symmetric probability density functions around $\omega ^*$, $f(\omega ^*-\delta ) = f(\omega ^* + \delta )$ and so for $\delta = \omega ^* - x$, we have $f(y)=f(x)$. Applying this transformation to the following integral yields

46 $$\begin{aligned} \mathbb {E}(d_\textrm{tr}(2\omega ^* - X,\omega ))&= \int _{\mathbb {R}^e/\mathbb {R}{} \textbf{1}} d_\textrm{tr}(2\omega ^* - x,\omega ) f(x) \, dx \nonumber \\&= \int _{\mathbb {R}^e/\mathbb {R}{} \textbf{1}} d_\textrm{tr}(y,\omega ) f(y) \, dy = \mathbb {E}(d_\textrm{tr}(X,\omega )). \end{aligned}$$

Combining Eq. (46) with inequality (45) shows that the function $Q(\omega ) = \mathbb {E}(d_\textrm{tr}(X,\omega ))$ has a global minimum at $\omega ^*$.

From Theorem 4 (uniform law of large numbers), set $f(x,\omega ) = d_\textrm{tr}(x,\omega )$ and observe that $f(x,\omega )$ is always continuous w.r.t. $\omega$. Setting $r(x) = \sup _{\omega \in \Omega } d_\textrm{tr}(x,\omega )$, which is finite since $\Omega$ is compact, observe that

$$\begin{aligned} r(x):= \sup _{\omega \in \Omega } d_\textrm{tr}(x,\omega ) \le d_\textrm{tr}(x,\omega ^*) + \sup _{\omega \in \Omega } d_\textrm{tr}(\omega ,\omega ^*). \end{aligned}$$

Since $\Omega$ is compact, the second term is finite and hence *r*(*X*) is integrable, since $d_\textrm{tr}(X,\omega ^*)$ is integrable. All conditions of the theorem are satisfied so $Q(\omega ) = \mathbb {E}(d_\textrm{tr}(x,\omega ))$ is continuous with respect to $\omega$ and

$$\begin{aligned} \sup _{\omega \in \Omega } |Q_n(\omega ) - Q(\omega )| \overset{p}{\rightarrow } 0 \text { as } n \rightarrow \infty , \end{aligned}$$

where $Q_n(\omega ) = n^{-1} \sum _{i=1}^n d_\textrm{tr}(X_i,\omega )$. Since $Q(\omega )$ has a unique minimum at $\omega ^*$, all conditions of Theorem 3 are satisfied and so $\tilde{\omega }_n \rightarrow \omega ^*$ as $n \rightarrow \infty$. $\square$

### Proof of Proposition 8

i. If $\omega - X_i$ has a unique maximum $M_i = {{\,\mathrm{arg\,max}\,}}_j\{\omega _j - (X_i)_j\}$ and unique minimum $m_i = {{{\,\mathrm{arg\,min}\,}}}_{j}\{\omega _j - (X_i)_j\}$, then the gradient is
  47 $$\begin{aligned} (\nabla f(x))_j = |\{i: M_i=j\}| - |\{i: m_i=j\}|. \end{aligned}$$
  For the converse, assume that the gradient is well-defined. From Eqs. (23)–(24) and following the first few sentences of Lemma 2
  $$\begin{aligned} d_\textrm{tr}(x+\epsilon E_j,y) + d_\textrm{tr}(x-\epsilon E_j,y) - 2d_\textrm{tr}(x,y) = \epsilon s_j(x-y), \end{aligned}$$
  where $s_j$ is defined in Eq. (26) of Lemma 2. Consequently,
  $$\begin{aligned} f(x+\epsilon E_j) + f(x- \epsilon E_j) - 2f(x) = \epsilon \sum _{i=1}^n s_j(X_i - \omega _i) \end{aligned}$$
  Since *f* has a well-defined gradient, $\sum _{i=1}^n s_j(X_i - \omega ) = 0$ i.e. $s_j(X_i - \omega ) = 0$ for all $(i,j) \in [n] \times [e]$. This can only happen iff $X_i - \omega$ has unique maximum and minimum component for all $i \in [n]$.
ii. Using Eq. (47), the gradient of *f* vanishes at $x=\omega$ if and only if
  48 $$\begin{aligned} |\{i: M_i=j\}| = |\{i: m_i=j\}|. \end{aligned}$$
  Moreover,
  $$\begin{aligned} f(\omega +v)&= \sum _{i=1}^n \max _k \left\{ \omega _k - (X_i)_k + v_k \right\} - \min _k \left\{ \omega _k - (X_i)_k + v_k \right\} \\&\ge \sum _{i=1}^n \omega _{M_i} - (X_i)_{M_i} + v_{M_i} - \omega _{m_i} + (X_i)_{m_i} - v_{m_i} \\&= f(\omega ) + \sum _{i=1}^n v_{M_i} - v_{m_i} \end{aligned}$$
  Finally, note that because of Eq. (48),
  $$\begin{aligned} \sum _{i=1}^n v_{M_i}&= \sum _{j=1}^e v_j |\{i \in [n]:M_i=j\}| \\ {}&\overset{\textrm{A33}}{=} \sum _{j=1}^e v_j |\{i \in [n]:m_i=j\}| = \sum _{i=1}^n v_{m_i}, \end{aligned}$$
  and so $f(\omega +v) \ge f(\omega )$ for all $v \in \mathbb {R}^e/\mathbb {R}{} \textbf{1}$.

$$\square $$

## Appendix B: Space of Ultrametrics

### Theorem 5

(explained in Ardila and Klivans (2006), Page et al. (2020)) Suppose we have a *classical* linear subspace $L_m \subset \mathbb {R}^e$ defined by the linear equations $x_{ij} - x_{ik} + x_{jk}=0$ for $1\le i< j <k \le m$. Let $\text{ Trop }(L_m)\subseteq \mathbb {R}^e/\mathbb {R}\textbf{1}$ be the *tropicalization* of the linear space $L_m \subset \mathbb {R}^e$, that is, classical operators are replaced by tropical ones (defined in Section C in the “Appendix”) in the equations defining the linear subspace $L_m$, so that all points $(v_{12},v_{13},\ldots , v_{m-1,m})$ in $\text {Trop}(L_m)$ satisfy the condition that

$$\begin{aligned} \max _{i,j,k\in [m]}\{v_{ij},v_{ik},v_{jk}\}. \end{aligned}$$

is attained at least twice. Then, the image of $\mathcal {U}_m$ inside of the tropical projective torus $\mathbb {R}^e/\mathbb {R}\textbf{1}$ is equal to $\text {Trop}(L_m)$.

## Appendix C: Tropical Arithmetics and Tropical Inner Product

In tropical geometry, addition and multiplication are different than regular arithmetic. The arithmetic operations are performed in the max-plus tropical semiring $(\,\mathbb {R} \cup \{-\infty \},\oplus ,\odot )\,$ as defined in Pin (1998).

### Definition 5

(*Tropical Arithmetic Operations*) In the tropical semiring, the basic tropical arithmetic operations of addition and multiplication are defined as:

$$\begin{aligned} a \oplus b:= \max \{a, b\}, ~~~~ a \odot b:= a + b, ~~~~ \text{ where } a, b \in \mathbb {R}\cup \{-\infty \}. \end{aligned}$$

The element $-\infty$ ought to be included as it is the identity element of tropical addition. Tropical subtraction is not well-defined and tropical division is classical subtraction.

The following definitions are necessary for the definition of the tropical inner product

### Definition 6

(*Tropical Scalar Multiplication and Vector Addition*) For any scalars $a,b \in \mathbb {R}\cup \{-\infty \}$ and for any vectors $v,w \in (\mathbb {R}\cup \{-\infty \})^e$, where $e \in \mathbb {N}$,

$$\begin{aligned} a \odot&v:= (a + v_1, \ldots ,a + v_e), \\ a \odot v \oplus b \odot w&:= (\max \{a+v_1,b+w_1\}, \ldots , \max \{a+v_e,b+w_e\}). \end{aligned}$$

From the definitions above, it follows that the tropical inner product is $\omega ^T \odot x = \max \{ \omega + x \}$ for all vectors $\omega ,x \in \mathbb {R}^e/\mathbb {R}{} \textbf{1}$. In classical logistic regression a linear function in the form of a classical inner product $h_{\omega }(x) = \omega ^T x, \, \omega \in \mathbb {R}^n$ is used. The tropical symbolic equivalent is

49 $$\begin{aligned} h_{\omega }(x) = \omega ^T \odot x = \max _{l \in [e]} \{ \omega _l + x_l \}. \end{aligned}$$

This expression is not well-defined, since the statistical parameter and covariate vectors $\omega , u \in \mathbb {R}^e / {\mathbb {R}} \textrm{1}$ are only defined up to addition of a scalar multiple of the vector $(1,\dots ,1)$. To resolve this issue, we fix

50 $$\begin{aligned} - \min _{l \in [e]} \{ \omega _l + x_l \} = c, \end{aligned}$$

where $c \in \mathbb {R}$ is a constant for all observations. Combining Eqs. (50), (49), and the definition of tropical distance (1),

$$\begin{aligned} h_{\omega }(x) = d_\textrm{tr}(x,-\omega ) - c. \end{aligned}$$

For simplicity, under the transformation $-\omega \rightarrow \omega$ the expression becomes

51 $$\begin{aligned} h_{\omega }(x) = d_\textrm{tr}(x,\omega ) - c. \end{aligned}$$

## Appendix E: Fermat–Weber Point Visualization

As noted in Sect. 3, the gradient method is much faster than linear programming. Unfortunately, there is no guarantee that it will guide us to a Fermat–Weber point. However, in practice, the gradient method tends to work well. Figure 10 illustrates just that. Given, ten datapoint $X_1, \dots , X_{10} \in \mathbb {R}^3/\mathbb {R}{} \textbf{1} \cong \mathbb {R}^2$, the Fermat–Weber set is found to be a trapezoid. This is in agreement with (Lin and Yoshida 2018), which states that all Fermat–Weber sets are classical polytopes. The two-dimensional gradient vector, plotted as a vector field in Fig. 10, always points towards the Fermat–Weber set. Therefore, the gradient algorithm should always guide us to a Fermat–Weber point.

## Appendix F: MLE Estimator for $\sigma$

If $Z_i \overset{\textrm{iid}}{\sim }\ \textrm{Gamma}(n, k)$, where *n* is constant and *k* is a statistical parameter, then it is well-known that the maximum likelihood estimator is

52 $$\begin{aligned} \hat{k} = \bar{Z}/n, \end{aligned}$$

where $\bar{Z}$ is the sample average. In our case $Z_i = d(X_i,\omega ^*)$ and $k= i \sigma ^i$. From Proposition 4, $Z_i \sim \textrm{Gamma}(n/i,i\sigma ^i)$ and by substituting these parameters in Eq. (52), it follows that the MLE for $\sigma$ is

$$\begin{aligned} \hat{\sigma }^i = \bar{Z} / n, \end{aligned}$$

where $\bar{Z}$ is the average distance of the covariates (gene trees) from their mean (species tree). This results holds for all $i \in \mathbb {N}$ and both Euclidean and tropical metrics. The only difference is that for Euclidean spaces $X \in \mathbb {R}^e$ and so $n=e$, while for the tropical projective torus $\mathbb {R}^e/\mathbb {R}{} \textbf{1}$, $n=e-1$.

## Appendix G: Approximate BHV Logistic Regression

Similar to the tropical Laplace distribution, in Billera et al. (2001) the following distribution was considered

$$\begin{aligned} f_{\lambda ,\omega }(x) = K_{\lambda ,\omega } \exp \left( -\lambda d_\textrm{BHV}(x,\omega ) \right) , \end{aligned}$$

where $\lambda = 1/\sigma$ is a concentration/precision parameter, $d_\textrm{BHV}$ is the BHV metric and $K_{\lambda ,\omega }$ is the normalization constant that depends on $\lambda$ and $\omega$. We consider an adaptation of the two-species model for this metric, where the data from the two classes have the same concentration rate but different centre. If $X|Y \sim f_{\lambda ,\omega ^*_Y}$, then

53 $$\begin{aligned} h_{\omega _0,\omega _1}(x) = \lambda \left( d_\textrm{BHV}(x,\omega _0^*) - d_\textrm{BHV}(x,\omega _1^*) \right) + \log {\frac{K_{\lambda ,\omega ^*_0 }}{K_{\lambda ,\omega ^*_1}}}. \end{aligned}$$

Unlike in the tropical projective torus or the euclidean space, in the BHV space $K_{\lambda ,\omega ^*_0 } \ne K_{\lambda ,\omega ^*_1}$, because the space is not translation-invariant. However, if we assume that the two centres are far away from trees with bordering topologies, it may be assumed that the trees are mostly distributed in the Euclidean space and as a result $K_{\lambda ,\omega ^*_0 } \approx K_{\lambda ,\omega ^*_1}$. Under this assumption, Eq. (53) becomes

$$\begin{aligned} h_{\omega _0,\omega _1}(x) \approx \lambda \left( d_\textrm{BHV}(x,\omega _0^*) - d_\textrm{BHV}(x,\omega _1^*) \right) . \end{aligned}$$

Therefore, the classification/decision boundary for the BHV is the BHV bisector $d_\textrm{BHV}(x,\omega _0^*) = d_\textrm{BHV}(x,\omega _1^*)$ and the most sensible classifier is

$$\begin{aligned} C(x) = \mathbb {I}\left( d_\textrm{BHV}(x,\omega _0^*) > d_\textrm{BHV}(x,\omega _1^*)\right) , \end{aligned}$$

where $\mathbb {I}$ is the indicator function.

## Appendix H: Graphs for Simulated Data under the Multi-species Coalescent Model for Different *R*

See Figs. 11 and 12.
